# Primary and Secondary Symbionts of Cambodian Cicadellidae and the Role of Parasitisation

**DOI:** 10.1111/1758-2229.70196

**Published:** 2025-09-16

**Authors:** Sophany Phauk, Lorenzo Assentato, Seanghun Meas, Olle Terenius

**Affiliations:** ^1^ Department of Cell and Molecular Biology, Microbiology Uppsala University Uppsala Sweden; ^2^ Department of Biology, Faculty of Science Royal University of Phnom Penh Phnom Penh Cambodia

**Keywords:** 16S rRNA, bacterial community, Cicadellidae, parasitism, *Sulcia*, symbiont

## Abstract

Leafhoppers (Hemiptera: Cicadellidae) are important vectors of plant pathogens in agricultural systems. Biological control via parasitisation is a key management strategy, but little is known about how microbial symbionts mediate host‐parasitoid interactions. Here, we characterise the bacterial communities of six Cambodian leafhopper species (*Cofana spectra*, *Exitianus* sp., *Goniagnathus punctifer*, *Maiestas dorsalis*, *Nephotettix virescens*, and *Stirellus* sp.) and their parasitoids from the families Dryinidae (Hymenoptera) and Halictophagidae (Strepsiptera). We found that the bacterial symbiont *Sulcia* dominates cicadellid microbiotas, often coexisting with secondary symbionts. For example, *Nasuia* is present alongside *Sulcia* in *Nephotettix*, while *Wolbachia* is prevalent in *Exitianus* and *Goniagnathus*. Parasitoids exhibited distinct microbiotas with greater diversity; Rhodobacteraceae and Comamonadaceae were in dryinids, while *Wolbachia* was common in Halictophagidae. We analysed the microbiota of individual pairs of host‐parasitoid and although parasitism did not significantly alter cicadellid overall microbiotas, some secondary symbionts (e.g., *Arsenophonus*, *Wolbachia*, *Rickettsia*, and *Sodalis*) were detected in both hosts and parasitoids, suggesting possible microbial transmission that warrants further investigation. These findings improve our understanding of host‐parasitoid microbial interactions and highlight the relationship between primary and secondary symbiont communities.

## Graphical abstract

Leafhoppers are key agricultural pests, and their interactions with parasitoids may be influenced by microbial symbionts. In this study, we characterized the bacterial communities of six Cambodian leafhopper species and their parasitoids, finding that while *Sulcia* dominates leafhopper microbiotas, parasitoids exhibit higher microbial diversity. Shared symbionts like *Wolbachia* and *Arsenophonus* were detected in both hosts and parasitoids, suggesting possible ecological overlap or symbiont transfer that merits further investigation.

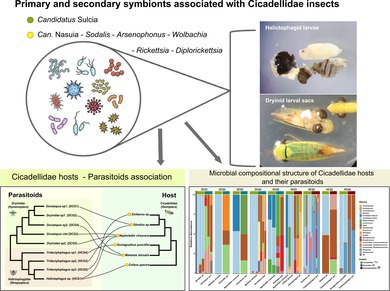

## 1 Introduction

Rice leafhoppers and planthoppers are major insect pests due to their economic and ecological impact (Baldridge and Blocker 1980; Cabauatan et al. 2009; Pathak and Khan 1994). Leafhoppers (Hemiptera: Cicadellidae) are a diverse and widespread group of phytophagous insects that vector microorganisms responsible for plant diseases (Baldridge and Blocker 1980). For instance, the green leafhoppers *Nephotettix malayanus* and *N. virescens* transmit viral diseases such as tungro virus and rice yellow dwarf virus (Cambodia, GDA 2015; Kay and Brown 1992; Rosida et al. 2020). A rise in green leafhopper populations can lead to substantial rice crop losses due to viral infections. These pests can be managed through modified agricultural practices, pesticide application (Pathak and Khan 1994) and biological control by natural enemies such as predators and parasitoids (Baldridge and Blocker 1980; van Lenteren 1983).

Parasitism is a form of symbiosis in which one organism (the parasite) lives on or inside a host, deriving nutrients at its expense, usually without killing the host (Balashov 2006; Root 1924). Parasites can play a significant role in the ecology and evolution of their hosts (Tseng and Myers 2014). In contrast, parasitoids, a distinct group of parasitic organisms (particularly among insects), complete their development by ultimately killing the host (Hughes et al. 2004). Due to their high specificity and lethal effects on hosts, parasitoids are wisely employed as effective biological control agents in diverse agroecosystems (van Lenteren 1983).

Among parasitoid arthropods, members of Dryinidae (Hymenoptera) and Halictophagidae (Strepsiptera) families are commonly associated with leafhoppers (Baldridge and Blocker 1980; Kathirithamby et al. 2003; McMahon et al. 2011; Pathak and Khan 1994; Virla et al. 2023). Dryinidae, also known as pincer wasps, are widespread parasitoids and predators of Auchenorryncha (Hemiptera), comprising over 1900 species (Virla et al. 2023). Baldridge and Blocker (1980) identified at least four species of Dryinidae parasitising 44 leafhopper species and five species of Halictophagidae parasitising 38 species. While dryinid parasitoids kill their hosts, strepsipteran parasitism (stylopization) is more prolonged and stable, often inducing significant morphological changes in hosts, such as extreme reduction or loss of external genitalia (McMahon et al. 2011).

While microbial symbioses in insects are increasingly well‐studied, their roles within host‐parasitoid interactions remain poorly understood. Arthropods harbour diverse microbial symbionts that influence biological functions, enhance host fitness, and provide protection against pathogens, parasitoids, and toxins (Gloder et al. 2021; Towett‐Kirui et al. 2023). Some intracellular symbionts reside within specialised bacteriocytes and play an essential role in insect evolution (Kucuk 2020; Rubin et al. 2018). A well‐known example is *Candidatus* Karelsulcia muelleri (hereafter *Sulcia*), a primary symbiont of Auchenorrhyncha (Cao and Dietrich 2021; Zchori‐Fein and Bourtzis 2011). Another example is the symbiotic bacterium *Wolbachia*, which is primarily known as a reproductive manipulator in arthropods but has also been reported to play nutritional roles in certain hosts (e.g., common bedbugs) (Hickin et al. 2022). It has been detected in both planthoppers (Delphacidae) and strepsipteran endoparasitoids (Hughes et al. 2004), raising the possibility of microbial exchange between hosts and parasitoids.

Microbial communities may also influence parasitic establishment both directly and indirectly, suggesting a three‐way interaction among the host, microbes, and parasitoid (Fredensborg et al. 2020). While host–microbe interactions have been well studied in free‐living insects, there is less research on microbes associated with parasitoids (Dheilly et al. 2019), both in endoparasitoids like Halictophagidae, which develop entirely within their host (Towett‐Kirui et al. 2023) and ectoparasitoids like Dryinidae, where larvae grow externally on the host (Virla et al. 2023). Recent studies on strepsipteran microbiota indicate that *Wolbachia* strains are widespread endosymbionts affecting arthropod reproduction and fitness, as seen in the tephritid fruit fly *Dipterophagus daci* (Towett‐Kirui et al. 2022; Towett‐Kirui et al. 2021). Similarly, microbial studies on the aphid *Aphis gossypii* and its parasitoid wasp *Lysiphlebia japonica* revealed that Proteobacteria, Firmicutes, and Actinobacteria dominate parasitoid bacterial communities (Gao et al. 2021). However, little is known about the bacterial composition of Dryinidae and Halictophagidae parasitoids or their interactions with Cicadellidae hosts.

In this study, we aim to characterise the bacterial communities of Cicadellidae hosts and their parasitoids (Dryinidae and Halictophagidae) using 16S rRNA gene amplicon Illumina sequencing. We conducted an in‐depth investigation of bacterial community composition in host‐parasitoid interactions, examining whether microbial taxa are shared between them and how these interactions influence cicadellid hosts, with a particular focus on endosymbionts. These results provide insight into the bacteria associated with cicadellid‐parasitoid interactions and contribute to a better understanding of their ecological roles.

## 2 Materials and Methods

### 2.1 Sites and Sample Collection

**FIGURE 1:**
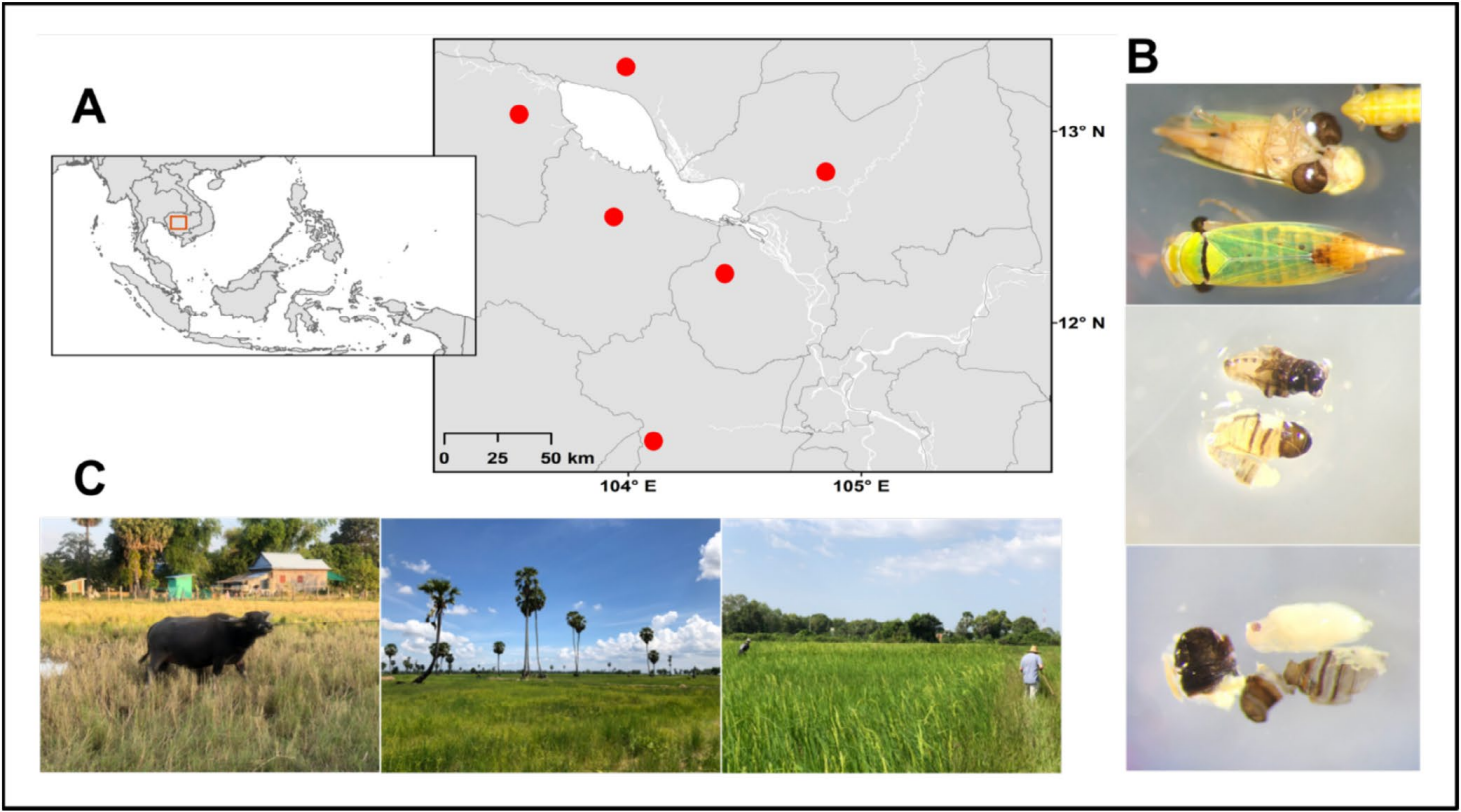
(A) Map showing the study sites, with collection locations highlighted in red. (B) Parasitoid‐host interaction, featuring cicadellid hosts (top), dryinid larvae (Hymenoptera) (middle) and halictophagid larvae (Strepsiptera) (bottom). (C) Examples of collection sites in paddy rice fields.

Samples were collected from the Chambok Community‐Based Ecotourism site (CBET), Kampong Speu province and five sites around the Tonle Sap Lake (TSL), Cambodia (Figure 1 and Table S1). The CBET habitats include paddy rice fields, bamboo, degraded semi‐evergreen and dipterocarp forest (Sin et al. 2020; Chhorn et al. 2020), while TSL is dominated by agricultural landscapes, primarily paddy rice fields. Insect sampling was conducted from July 2019 to September 2020. Insects were captured using sweep nets and transferred via aspirators into 1.5 mL Eppendorf tubes containing 95% ethanol for preservation. Specimens were deposited at the Cambodian Entomology Initiatives (CEI), Royal University of Phnom Penh, for sorting and identification.

### 2.2 Cicadellids and Parasites

Six cicadellid taxa—*Cofana spectra, Exitianus* sp., *Maiestas dorsalis, Nephotettix virescens*, *Stirellus* sp. and *Goniangnathus punctifer—*were identified based on morphological traits. Whenever identification was only possible to the genus level (*Exitianus* sp. and *Stirellus* sp.), all individuals were morphologically uniform and collected from the same habitat; these were therefore treated as single operational taxonomic units for this study. From the total field collection, individuals were screened for parasitism under the stereomicroscope, and 30 parasitised cicadellids identified by visible larval sacs of Dryinidae (ectoparasitic larvae) or internal larvae of Halictophagidae (endoparasitic larvae) were selected and dissected. Thirty non‐parasitised individuals were also chosen to match the host species composition of the parasitised group. Parasitoid larvae extracted from the parasitised hosts, along with their respective host, were used for molecular analysis, yielding 30 host‐parasitoid pairs. Only specimens with clear morphological identification and sufficient DNA concentration were retained for sequencing and microbial community analysis. We amplified and sequenced the Cytochrome C Oxidase subunit I (COI) gene using universal primers (LCO1490 and HCO2198) to assist in species‐level confirmation. Of the 30 parasitoid larvae, 23 were successfully assigned to Dryinidae or Halictophagidae based on NCBI and BOLD database matches (Figure S1). Seven samples were excluded due to low DNA concentration.

### 2.3 DNA Extraction

Samples were then transferred to Sweden for DNA extraction and amplification at the Department of Ecology, Swedish University of Agricultural Science (SLU) in Uppsala. DNA was extracted from a total of 90 whole‐tissue samples, including 30 non‐parasitised hosts, 30 parasitised hosts and 30 parasitoids (Table S1). Extractions were performed using the QIAamp DNA Mini Kit Protocol for Gram‐positive bacteria (Qiagen). To ensure data quality, two templates of microbial community standards (*Zymo*BIOMICs, Zymo Research, Irvine, CA, USA) were included as positive controls (Figure S2).

### 2.4 PCR Amplification

PCR amplification of extracted DNA samples (*n* = 83) was performed using a two‐step method (Buck et al. 2016) to generate barcoded bacterial 16S rRNA gene amplicons for sequencing (Table S1). The targeted 16S rRNA gene regions (V3‐V4) were amplified using bacterial primers 341F (5′‐CCT ACG GGN GGC WGC AG‐3′) and 805R (5′‐GAC TAC HVG GGT ATC TAA TCC‐3′) (Herlemann et al. 2011). Each PCR reaction contained 50–100 ng DNA as a template. The first PCR step conducted an initial denaturation at 95°C for 5 min, followed by 25 cycles of [95°C for 40 s, 53°C for 40 s and 72°C for 60 s], with a final elongation at 72°C for 7 min. For samples where DNA was not detected in the first run, the cycle number was increased to 35 cycles to improve amplification efficiency. The PCR products were analysed by using Gel Electrophoresis (1% TAE agarose gel) and DNA quantification was performed using Image Lab 6.0 software. The amplification DNA was then diluted in nuclease‐free water to a concentration of 0.1–1 ng μL^−1^ for the second PCR step (Nilsson et al. 2018).

In the second PCR step, 1 μL of diluted first‐step PCR product was used as a template. To enable multiplexing of up to 50 samples per sequencing pool, different barcoding primer pairs were applied (Table S5), following the method described by Sinclair et al. (2015). The second PCR following the same reaction conditions as the first step but with only 10 cycles. The resulting PCR products were pooled, purified and eluted in 50 μL nuclease‐free water. All PCR reactions were performed using illustra PuReTaq‐To‐Go PCR Beads (GE Health Care).

For parasitoid larvae (Dryinidae and Halictophagidae), a nested PCR protocol was used given the low DNA concentration in these samples. The first PCR step targeted the full‐length 16S rRNA gene using the universal primers 8F (5′‐AGA GTT TGA TCC TGG CTC AG‐3′) and 1505R (5′‐ACG GYT ACC TTG TTA CGA CTT‐3′) (Ku and Lee 2014). The same PCR cycling conditions were applied, but with 30 cycles instead of 25. The second PCR step was performed following the V3–V4 amplification protocol described above.

### 2.5 Library Preparation and MiSeq Sequencing

Sequencing libraries and MiSeq sequencing were prepared and performed by the SNP&SEQ Technology Platform at Uppsala University, Sweden (https://snpseq.medsci.uu.se/). Libraries were prepared with 5 ng of DNA input per sample using the SMARTer ThruPLEX DAN‐seq library preparation kit (cat# R400676, Takara) with unique dual index set A–D (cat# r400665/6/7/8, Takara). The library preparation was performed according to the manufacturers' instructions (guide#112219). Sequencing was performed by paired‐end sequencing with 300 bp read length and v3 sequencing chemistry using the MiSeq system (Illumina) according to the manufacturer's protocols. A sequencing library for the phage PhiX was included as 10% spike‐in in the sequence run.

### 2.6 Pre‐Processing of Bioinformatic Data

After retrieving the .*fastq* files corresponding to the pools from the sequencing facility, these were processed at the Uppsala Multidisciplinary Center for Advanced Computational Science (UPPMAX), provided by Swedish National Infrastructure for Computing (SNIC), project NAISS 2025/22‐339. The pools were demultiplexed through the use of *Cutadapt* (v. 4.1) (Martin 2011), searching for the pair of barcodes (‐pair‐adapters) corresponding to the study samples in both normal and reversed orientation, with default settings. The individual samples were then processed to remove the PCR primers, corresponding to the pair previously mentioned 341F‐805R. Primers were allowed 10% of errors in the sequence (1 error for the forward of 17 nt, 2 errors for the reverse of 21 nucleotide, respectively), and the pairs were allowed to pass a length filter only if both sequences were in the range of 200–300 nt, to mitigate any influence of non‐specific priming. Sequences where the primers were not found were discarded from the samples. Samples included in this study (at this stage of processing) have been deposited and are available in the European Nucleotide Archive (ENA) under the accession number PRJEB70784.

### 2.7 ASV Inference and Taxonomical Identification

The samples were then further processed, first through Trim Galore (v. 0.6.7) (Krueger et al. 2023), with default options to operate a variable threshold trimming based on quality. Afterwards, the samples were processed using the DADA2 package (v. 1.26.0) in R (v. 4.2.0) (Callahan et al. 2016), which operated a filtering of the reads using standard parameters, with no additional trimming since the reads had been trimmed before. As per the standard DADA2 workflow, after creating an error model for the run, amplicon sequence variants (ASVs) were inferred with a pooling algorithm. Reads were merged (still using DADA2, function ‘mergePairs’), and the results filtered by length, retaining only ASVs with a length between 350 and 470 nt; chimeric sequences were also removed using the standard DADA2 chimera removal implementation through the function ‘removeBimeraDenovo’ Taxonomic identification of ASVs was conducted with the DECIPHER package (v. 2.26.0) using IDTAXA (Wright 2016), on both strands, aligning to the modified SILVA SSU (r138) (Murali et al. 2018) training set.

The ASV count table, taxonomy table and ASV list were produced, subsequently the ASV sequences were used through *MAFFT* (v. 7.508) (Katoh and Standley 2013) to generate a multiple sequence alignment, with default parameters and automatic settings (−auto), the generated alignment file was then used to build an unrooted tree with the use of IQ‐Tree (v. 2.2.0.3) (Nguyen et al. 2015) using Model Finder Pro (‐MFP). The ASV count table, taxonomy table and the tree file were imported into *Phyloseq* (v. 1.42.0) (McMurdie and Holmes 2013), for further processing. In *Phyloseq* the ASVs were filtered, first removing any sequence that was unclassified at the highest taxonomical level (Phylum), then sequences belonging either to Chloroplasts or Mitochondria. The counts were also normalised using DESeq2 package (v. 1.38.0) (Love et al. 2014) applying a variance stabilised transformation (VST). Two *Phyloseq* objects were retained for further processing, one with normalised counts and one with absolute counts, everything has been saved in an .*RData* object.

### 2.8 Statistical Analysis

Downstream analyses were conducted in R (v. 4.3.2). A phyloseq object of the dataset was generated using the Phyloseq package (v. 1.4.2) (McMurdie and Holmes 2013) for further processing. Bacterial community composition across different sample types was analysed at the phylum, family, and genus levels, and visualised as stacked bar plots. The ggplot2 package (Wickham 2016) was used to generate bacterial composition visualisations (Figure 3). The analysis of host‐endosymbiont associations (Figure 4) and co‐occurrence of microbiota in the hosts and their parasitoids (Figure 5) was conducted using the ComplexHeatmap package (v. 2.14.0) (Gu 2022).

## 3 Results

### 3.1 Host–Parasite Interactions

**FIGURE 2:**
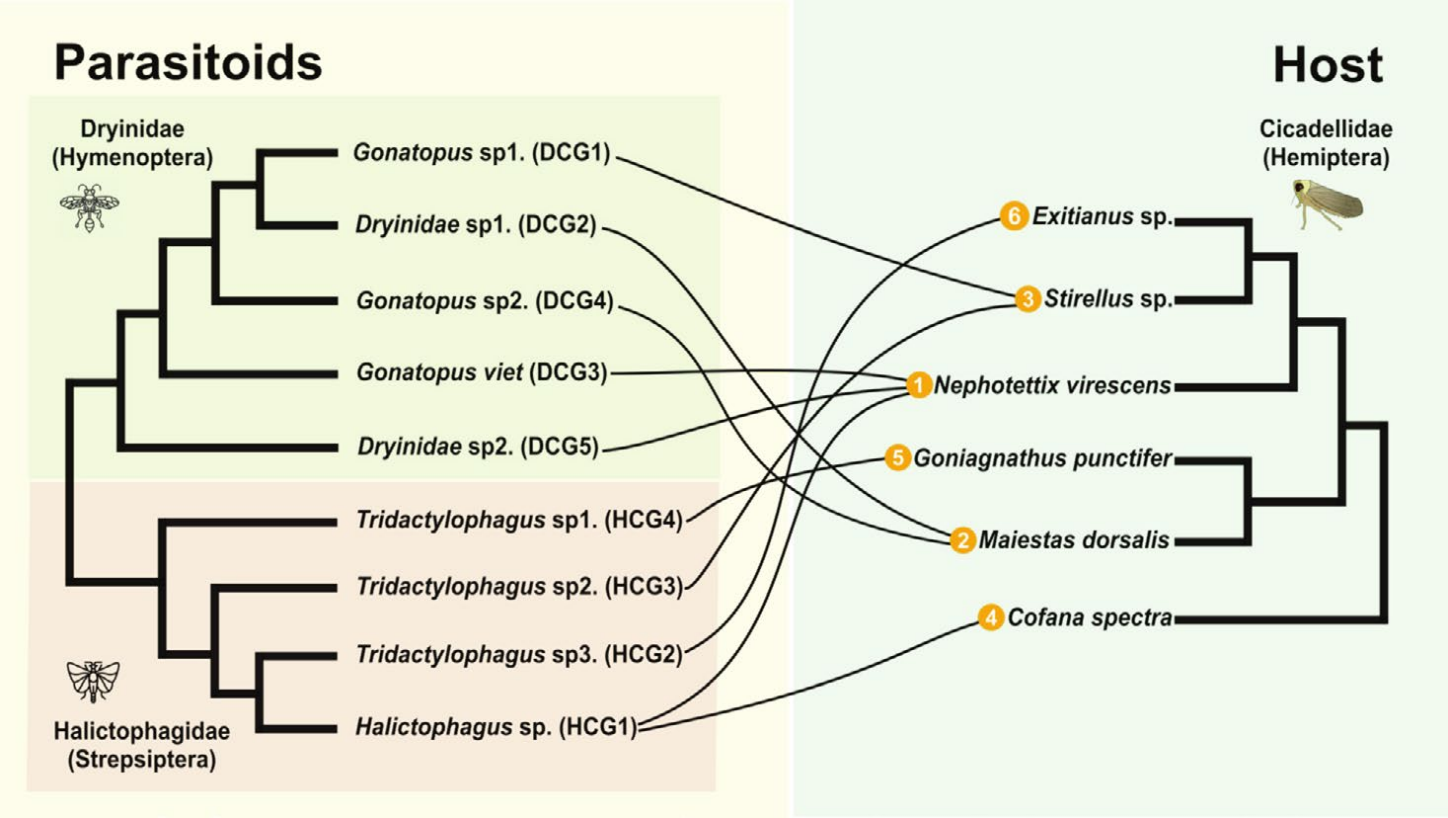
Schematic diagram of the interaction between hosts Cicadellidae and parasitoids (Drynidae and Halictophagidae). The numbers in the orange circles represent host‐parasitoid interaction groups. The diagram is based on the phylogenetic trees of maximum‐likelihood on COI gene sequences run with 1000 bootstraps.

In this study, we identified nine parasitic insect species (Dryinidae and Halictophagidae) parasitising six host species of Cicadellidae in Cambodia (Figures 2 and S1) based on dissected larvae. The phylogenetic tree of parasitoid insects resulted in nine groups of interaction with their respective host. Parasitoids were classified into five Dryinidae‐Cicadellidae groups (DCG) and four Halictophagidae‐Cicadellidae groups (HCG). We found that the Halictophagidae (HCG) groups parasitised five cicadellid hosts: *Cofana spectra, Nephotettix virescens*, *Exitianus* sp., *Goniangathus punctifer* and *Stirellus* sp. Meanwhile, the Dryinidae (DCG) groups were associated with three cicadellid hosts: *Maiestas dorsalis, Nephotettix virescens* and *Stirellus* sp. Notably, *Stirellus* sp. and *N. virescens* were parasitised by both Dryinidae and Halictophagidae.

### 3.2 ASV Assignment and Bacterial Community Structures

A total of 1,278,776 raw sequence reads were obtained from samples, including parasitised hosts, non‐parasitised hosts, and parasitoid larvae. After quality filtering and denoising using the DADA2 pipeline, 1,163,804 reads were retained, accounting for an average of 88.37% of the sample set (Table S2). Following taxonomic filtering, 897,550 sequence reads remained, leading to the identification of 364 ASVs. After taxonomic filtering, 64 ASVs, comprising sequences from chloroplasts and mitochondria, were removed, resulting in a final dataset of 300 ASVs.

### 3.3 Bacterial Composition in the Cicadellid Hosts and Parasitoids

**FIGURE 3:**
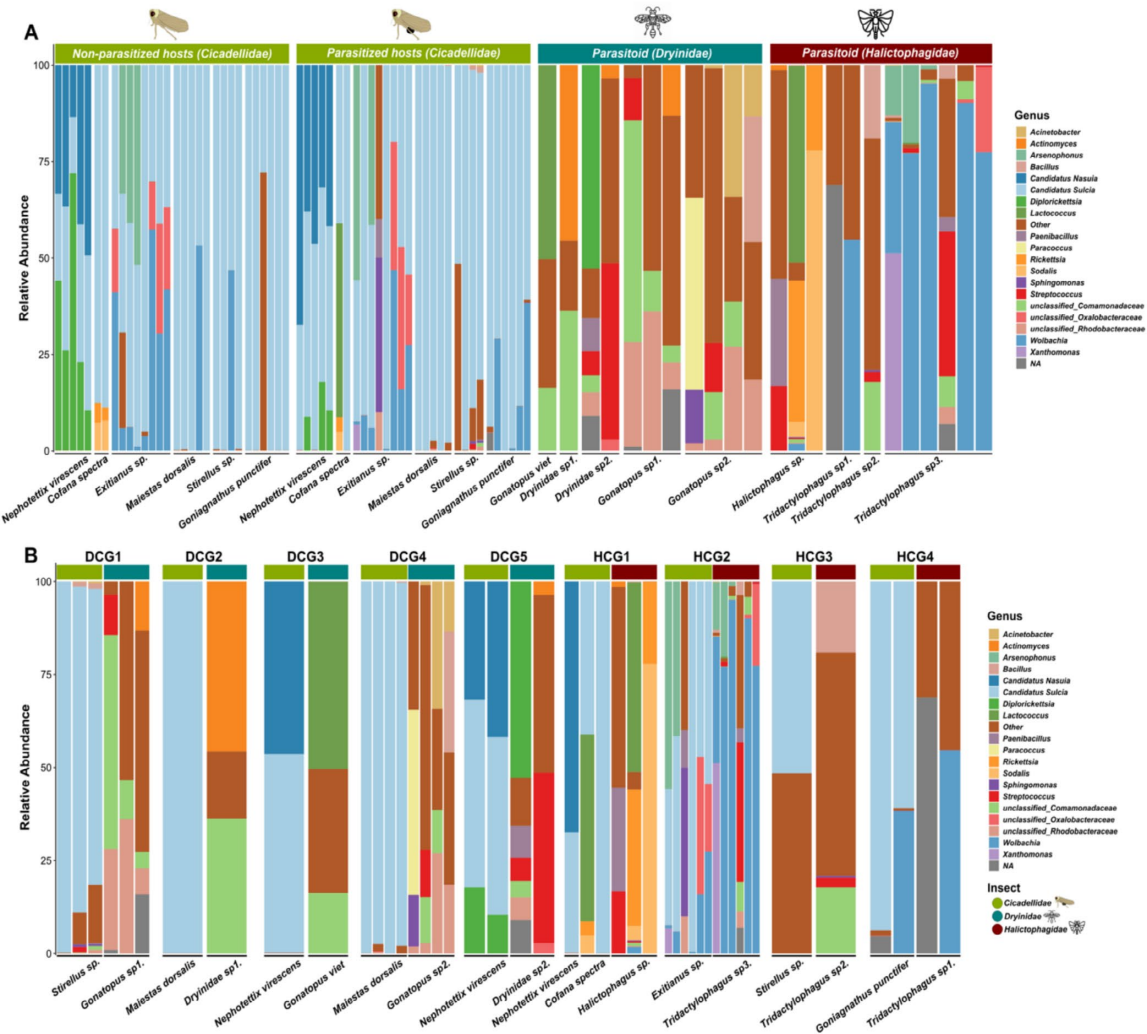
Bacterial composition of top 20 taxa at the genus level: (A) Cicadellidae (parasitised and non‐parasitised) hosts and their parasitoids (Dryinidae and Halictophagidae). (B) Bacterial compositional interaction between parasitised hosts and their parasitoids (DCG = Dryinidae‐Cicadellidae Group; HCG = Halictophagidae‐Cicadellidae Group). Each cluster represents a parasitised host and its associated parasitoid grouped by interaction pairings (e.g., DCG1 includes *Stirellus* sp. and its corresponding Dryinid parasitoid). The interaction pairings represent the composition of samples (species) corresponding to host‐parasitoid species interactions shown in Figure 2. ‘*NA*’ indicates unclassified bacterial taxa at the genus level.

Overall, we identified 11 bacterial phyla in the dataset: Abditibacteriota, Actinobacteriota, Bacteroidota, Campilobacterota, Cyanobacteria, Firmicutes, Fusobacteriota, Patescibacteria, Planctomycetota, Proteobacteria, and Verucomicrobiota (Figure S3). Among all Cicadellidae hosts (parasitised and non‐parasitised), the bacterial community was dominated by members of the phylum Bacteroidota, represented solely by the intracellular endosymbiont genus *Sulcia*, which accounted for 49.94% of the total sequences. In parasitised hosts, five bacterial phyla—Actinobacteriota, Bacteroidota, Cyanobacteria, Firmicutes, and Proteobacteria—were detected. The most dominant was Bacteroidota (70.87%) followed by Proteobacteria (29.63%). Bacterial composition characterised into seven classes, 24 orders, 40 families, and 49 genera. Bacteroidia was the most dominant class (70.87%), followed by Gamma‐Proteobacteria (15.6%) and Alpha‐Proteobacteria (10.95%). The most abundant families were Blattabacteriaceae (70.36%), Oxalobacteriaceae (10.47%) and Anaplasmataceae (6.18%). At the genus level, *Sulcia* (70.36%), *Nasuia* (7.51%), and *Wolbachia* (6.18%) were dominant, respectively (Figure 3A; Table 1).

**TABLE 1:** Summary of the relative abundance bacterial composition from Phylum to Genus level in the datasets and sample types.

Phylum	Class	Order	Family	Genus	Non‐parasitised host *Cicadellidae* (*N*: 30)	Parasitised host *Cicadellidae* (*N*: 30)	Parasitoid *Dryinidae* (*N*: 11)	Parasitoid *Halictophagidae* (*N*: 12)
Bacteroidota	Bacteroidia	Flavobacteriales	Blattabacteriaceae	Candidatus Sulcia	**67.823**	**70.366**	NA	NA
Proteobacteria	Alphaproteobacteria	Rickettsiales	Anaplasmataceae	Wolbachia	9.572	6.186	NA	**35.841**
Proteobacteria	Gammaproteobacteria	Burkholderiales	Oxalobacteraceae	Candidatus Nasuia	5.816	7.515	NA	NA
Proteobacteria	Gammaproteobacteria	Diplorickettsiales	Diplorickettsiaceae	Diplorickettsia	5.840	1.231	5.275	0.0016
Proteobacteria	Gammaproteobacteria	Enterobacterales	Morganellaceae	Arsenophonus	4.211	3.246	NA	2.8465
Proteobacteria	Gammaproteobacteria	Burkholderiales	Oxalobacteraceae	Unclassified_Oxalobacteraceae	2.617	2.951	0.254	1.9112
Proteobacteria	Gammaproteobacteria	Burkholderiales	Comamonadaceae	Unclassified_Comamonadaceae	0.001	0.038	**14.110**	2.7462
Firmicutes	Bacilli	Lactobacillales	Streptococcaceae	Lactococcus	0.000	1.676	4.576	4.2891
Proteobacteria	Alphaproteobacteria	Rhodobacterales	Rhodobacteraceae	Unclassified_Rhodobacteraceae	0.000	0.387	11.697	0.4323
Firmicutes	Bacilli	Lactobacillales	Streptococcaceae	Streptococcus	0.002	0.058	6.948	5.1569
Proteobacteria	Gammaproteobacteria	Enterobacterales	Pectobacteriaceae	Sodalis	0.500	0.160	NA	6.8196
Proteobacteria	Gammaproteobacteria	Enterobacterales	Erwiniaceae	Unclassified_Erwiniaceae	NA	0.051	NA	7.4743
Proteobacteria	Gammaproteobacteria	Piscirickettsiales	Piscirickettsiaceae	Candidatus Endoecteinascidia	2.406	0.001	NA	*NA*
Proteobacteria	Alphaproteobacteria	Rickettsiales	Rickettsiaceae	Rickettsia	0.282	0.128	NA	4.8958
Actinobacteriota	Actinobacteria	Actinomycetales	Actinomycetaceae	Actinomyces	NA	NA	5.899	0.1166
Firmicutes	Bacilli	Bacillales	Bacillaceae	Bacillus	0.004	0.102	2.966	1.9893
Proteobacteria	Gammaproteobacteria	Xanthomonadales	Xanthomonadaceae	Xanthomonas	0.001	0.223	NA	4.2644
Proteobacteria	Alphaproteobacteria	Sphingomonadales	Sphingomonadaceae	Sphingomonas	NA	1.376	1.258	0.0745
Firmicutes	Bacilli	Paenibacillales	Paenibacillaceae	Paenibacillus	0.000	0.345	0.858	2.6478
Proteobacteria	Alphaproteobacteria	Rhodobacterales	Rhodobacteraceae	Paracoccus	0.000	NA	4.531	NA
Proteobacteria	Gammaproteobacteria	Pseudomonadales	Moraxellaceae	Acinetobacter	0.002	0.023	4.413	0.0258
Proteobacteria	Alphaproteobacteria	Rickettsiales	Fokiniaceae	Candidatus Lariskella	0.004	1.582	NA	NA
				*Other bacteria* ^a^	0.918	2.354	37.214	18.466

*Note:* Top 22 bacterial taxa at the Genus level based on the relative abundance (%) in the dataset (*N* = 83). NA: absence of the bacterial taxon in the sample type. Bold indicates the dominant bacteria in sampled group.

^a^ *Other bacteria* include 68 taxa with less than 10% abundance in the overall dataset. These taxa are provided in Table S3.

The bacterial composition of Dryinidae (Hymenoptera) parasitoids included seven phyla, nine classes, 27 orders, 38 families and 50 genera. The seven phyla were classified as Actinobacteriota, Bacteroidota, Cyanobacteria, Firmicutes, Fusobacteriota, Patescribacteria and Proteobacteria. The abundant phyla were Proteobacteria (55.5%), Firmicutes (26.68%) and Actinobacteriota (15.45%). The most dominant classes were Gamma‐Proteobacteria (29.13%), Alpha‐Proteobacteria (26.37%), Bacilli (21.77%) and Actinobacteria (15.45%) respectively. At the family level, the most dominant groups were Rhodobacteraceae (16.03%), Comamonadaceae (14.09%), Streptococcaceae (11.45%), Sphingomodaceae (7.21%) and Actinomycetaceae (6.11%). The dominant genera were unclassified Comamondaceae (14.11%) and unclassified Rhodobacteraceae (11.70%) followed by *Streptoccoccus* (6.95%), *Actinomyces* (5.90%) and *Diplorickettsia* (5.27%) (Figure 3).

For Halictophagidae (Strepsiptera) parasitoids, bacterial composition classified into nine phyla, 12 classes, 33 orders, 51 families and 65 genera. The nine phyla were Abditibacteriota, Actinobacteriota, Bacteroidota, Campilobacterota, Firmicutes, Fusobacteriota, Planctomycetota, Proteobacteria and Verrucomicrobiota. The most abundant phyla were Proteobacteria (77.56%) and Firmicutes (18.91%). At the class level, Alpha‐Proteobacteria was the most dominant (41.54%), followed by Gamma‐Proteobacteria (36.02%) and Bacilli (16.38%). The most abundant families were Anaplasmataceae (35.84%), Streptococcaceae (9.21%), Pectobacteriaceae (6.82%), Xanthomonadaceae (5.85%) and unclassified Enterobacterales (5.73%). At the genus level, *Wolbachia* was the most dominant at the 35.84% coverage and followed by unclassified Erwiniaceae (7.47%), *Sodalis* (6.82%) and *Streptococcus* (5.15%) (Figure 3A).

### 3.4 Cicadellid Hosts—Endosymbiont Associations

**FIGURE 4:**
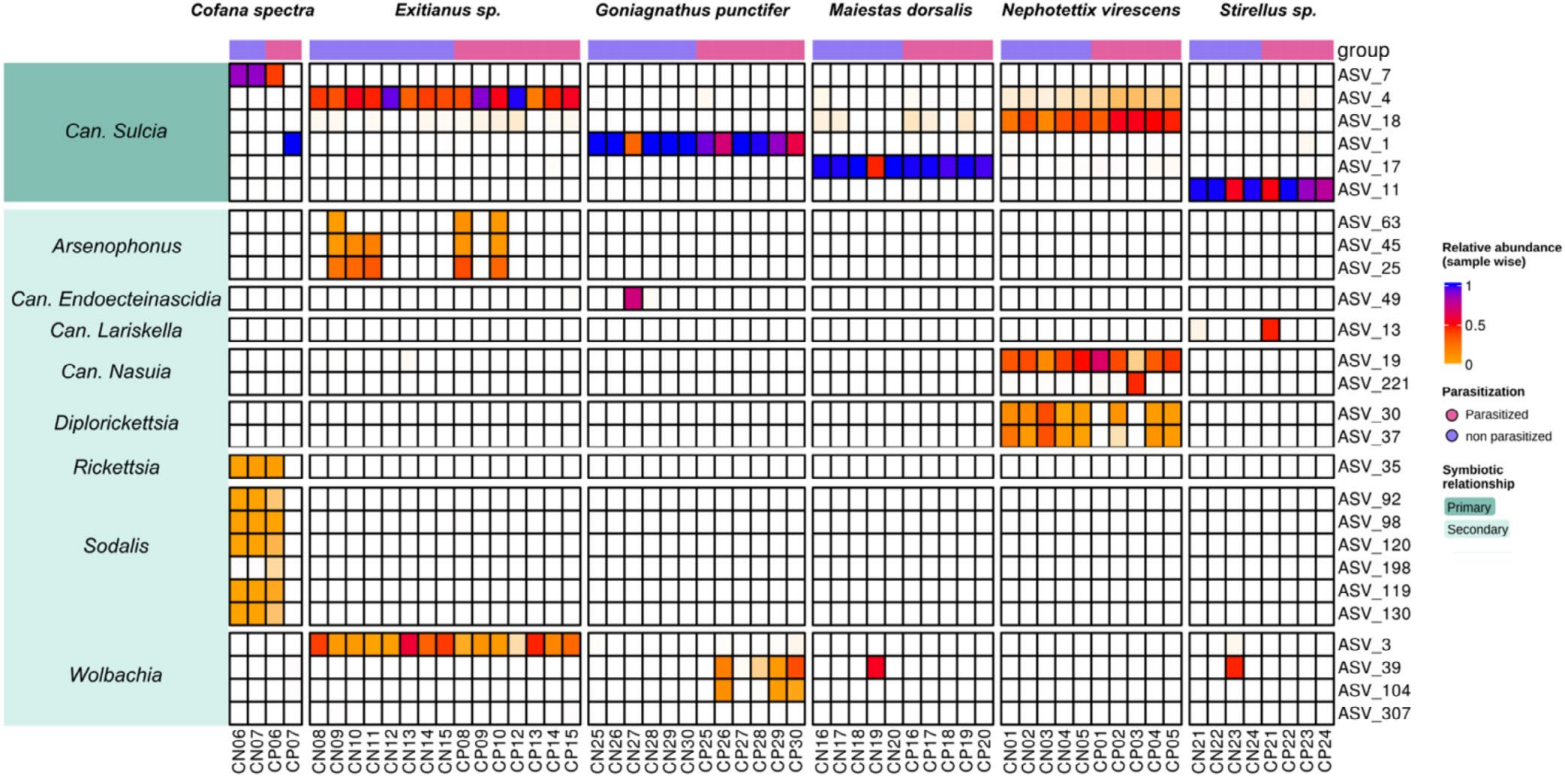
Heatmap of common endosymbionts associated with Cicadellidae hosts: The heatmap shows ASVs of primary/secondary symbionts for Cicadellidae and their relative abundance (calculated sample‐wise on the total number of reads per sample). To avoid the presence of singletons and low‐abundance ASVs, the lower limit of the scale has been set to 0.01 (1%) of relative abundance.

The obligate endosymbiont *Sulcia* was the most dominant bacterium in cicadellid hosts. While *Sulcia* was consistently present across cicadellid hosts (with several identified ASVs), the composition of co‐primary and secondary symbionts varied significantly between species (Figure 4; Table S4). We identified in most cases two secondary symbionts in cicadellid hosts, except for *Maiestas dorsalis*, which harboured only Wolbachia, and only in a single specimen. In *Cofana spectra*, both *Sodalis* and *Rickettsia* were detected as secondary symbionts. Additionally, Goniagnathus punctifer **harboured** Wolbachia and Can. Endoecteinascidia, while *Stirellus* sp. hosted Wolbachia and Can. Lariskella, only in two instances, but in separate individuals. Notably, within the tribe **Chiasmini**, *Exitianus* sp. and Nephotettix virescens harboured distinct secondary symbionts. Exitianus sp. carried Arsenophonus and Wolbachia, whereas N. virescens was associated with Nasuia (co‐primary) and *Diplorickettsia*. Overall, *Wolbachia* was detected in most cicadellid host species, with the exception of *N. virescens* and *C. spectra*.

### 3.5 Co‐Occurrence of Bacterial ASVs in Parasitised Hosts and Parasites

**FIGURE 5:**
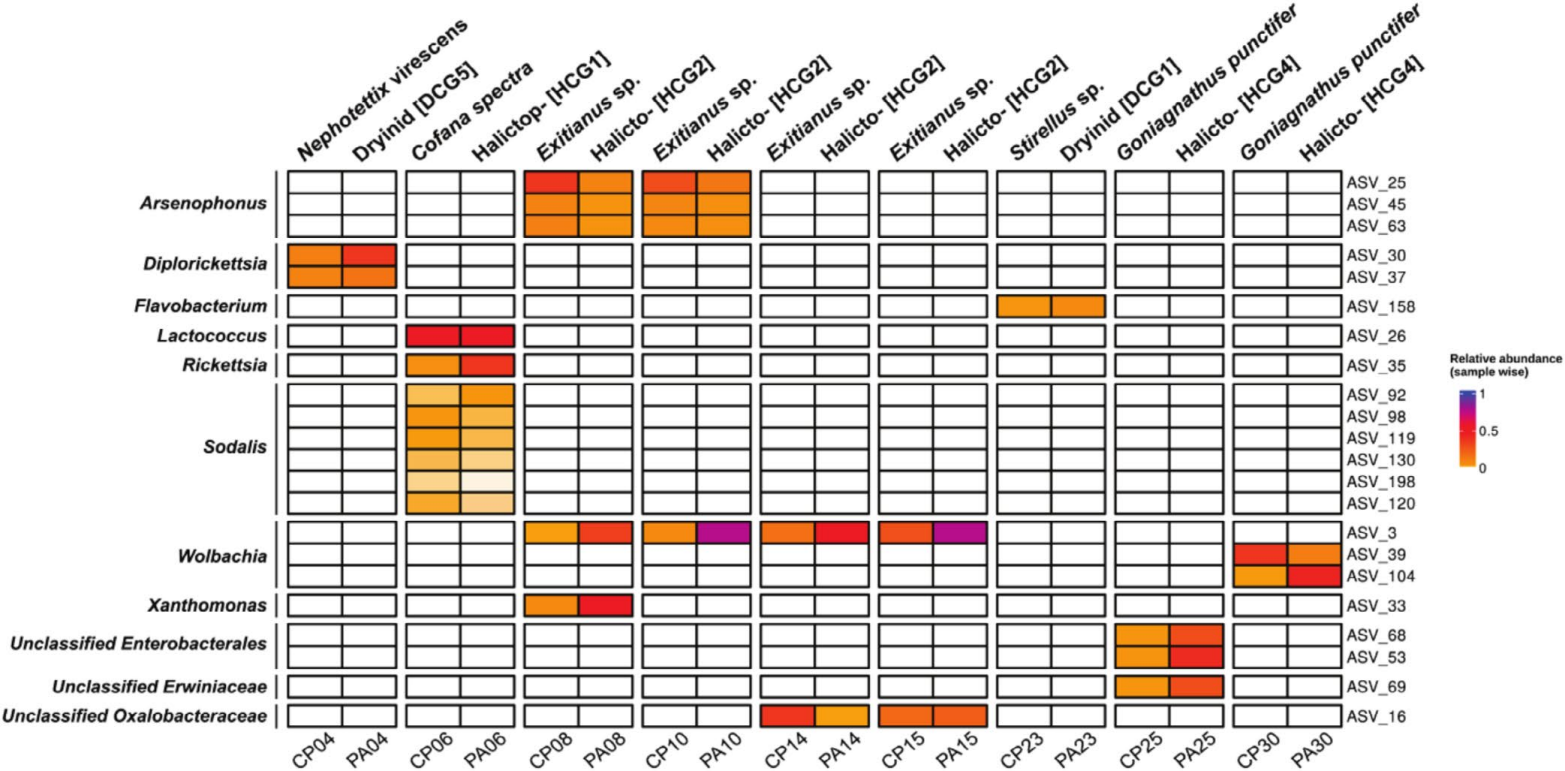
Bacterial co‐occurrence between individual hosts and parasitoids: Heatmap showing relative abundance (calculated sample‐wise on the total number of reads per sample) of selected ASVs and host‐parasitoid pairings. Pairs were selected if host and parasitoid samples shared any ASV. In addition to that, ASVs were selected only if the abundance of that ASV in the pair exceeds 50 reads. To avoid the presence of low‐abundance ASVs, the lower limit of the scale has been set to 0.01 (1%) of relative abundance. Hosts are positioned in the left column of each pair (CP), with the associated parasitoids on the right (PA).

To explore potential microbial transmission between parasitoids and their parasitised hosts, we examined co‐occurrence patterns. ASVs that are present in both the parasitoid and the corresponding host are summarised in Figure 5. Despite many pairs of parasitoids and hosts not sharing ASVs (not shown in the figure), there are some notable exceptions. In particular, *Exitianus* sp. and the associated parasitoid group Halictophagidae (HCG2) are represented by four different parasitoid‐host pairs. For this instance, three ASVs from *Arsenophonus* were found in both members of the pair, although the co‐occurrence happened only in two out of four parasitoid‐host pairs of this kind (Figure 2, interaction group 6). The behaviour observed for ‘ASV_3’ of *Wolbachia* is also interesting, where instead we see a consistent presence in parasitoid‐host pairings, in four out of four pairs of the same interaction group. Still in the context of the same interaction group, we can also observe an ASV of *Oxalobacteraceae* being shared in two out of four pairs, as well as an ASV of *Xanthomonas* being shared in one out of four pairs. Two ASVs of *Diplorickettsia* are shared between *Nephotettix virescens* and Dryinidae [DCG5] (Figure 2, interaction group 1). *Cofana spectra* and Halictophagidae [HCG1] (Figure 2, interaction group 4) share a single ASV of *Lactococcus* and *Rickettsia*, as well much as six ASVs of *Sodalis*. *Stirellus* and *Gonatopus* [DCG1] (Figure 2, interaction group 3) share only an ASV of *Flavobacterium*. *Goniagnathus punctifer* and Halictophagidae [HCG4] (Figure 2, interaction group 5) share two ASVs of *Enterobacterales*, one of *Erwiniaceae* in one out of the two pairs, while in the other existing pair they share two *Wolbachia* ASVs.

## 4 Discussion

In this study, we investigated the interactions between different species of the family Cicadellidae and their parasitoids, Dryinidae and Halictophagidae from Cambodia. Significant differences in bacterial community structure were found among Cicadellidae hosts and both parasitoid insects, as well as host–parasite interaction groups (Figure 3B). These differences in bacterial community patterns across different species and groups may be influenced by several factors, including host‐specific traits, environmental conditions, and evolutionary relationships (Douglas 2015; Engel and Moran 2013). The results focused on microbial composition and the potential influence of parasitisation on symbiont distribution, as well as characterising interactions between hosts and both primary and secondary symbionts. Overall, parasitisation by Dryinidae and Halictophagidae did not lead to significant shifts in the overall bacterial community composition of *Cicadellidae* hosts when compared to non‐parasitised individuals. Regardless, along the dominant obligate endosymbiont *Sulcia*, multiple secondary symbionts were identified, and in some instances their presence was exclusive to parasitised individuals.

### 4.1 Primary and Secondary Endosymbionts

Using 16S rRNA gene amplicon sequencing, we characterised the bacterial communities in Cicadellidae and their parasitoid insects. A notable feature of the Cicadellidae microbiota was its low taxonomic complexity, consistent with the pattern observed in other sap‐feeding insects whose nutritionally unbalanced diets often support simplified bacterial communities. By contrast, parasitoid insects exhibited greater microbial diversity (Moran et al. 2005; Moriyama et al. 2023).

The obligate endosymbiont ***Sulcia*** was present in all Cicadellidae samples, confirming previous findings across sap‐feeding Auchenorrhyncha insects, due to their sap‐feeding behaviour and playing a crucial role in providing nutrients to Cicadellidae hosts (Cao and Dietrich 2021; Cooper et al. 2023; Ishii et al. 2013; Kobiałka et al. 2016, 2020; Kobiałka, Michalik, Szwedo, and Szklarzewicz 2018; Kobiałka, Michalik, Walczak, and Szklarzewicz 2018; Wu et al. 2023). Cao and Dietrich (2021) found that 73 of 96 auchenorrhynchan insects harboured *Sulcia*. Typically, *Sulcia* synthesises eight of the ten essential amino acids missing from the host's diet (Bennett and Moran 2013; Huang et al. 2023; Moran et al. 2005; Moriyama et al. 2023), with a co‐obligate endosymbiont supplementing the rest. One example is *Nasuia*, previously detected in several Deltocephalinae species (Kobiałka, Michalik, Szwedo, and Szklarzewicz 2018), including *Nephotettix cincticeps* (Moriyama et al. 2023; Noda et al. 2012), *Matsumuratettix hiroglyphicus* (Wangkeeree et al. 2012), and *Macrosteles striifons* and *M. sexnotatus* (Ishii et al. 2013). We detected *Nasuia* only in Nephotettix virescens, along with two ASVs of *Diplorickettsia* (Figure 4). *Nasuia* and *Sulcia* appear to be complementary, suggested also by comparable abundances. Moreover, in ‘CP03’, when the abundance of an ASV of *Nasuia* (ASV_19) is reduced, a different *Nasuia* ASV (ASV_221) is also present. At the same time, *Diplorickettsia* is absent from CP03, suggesting a potential functional complementarity between *Diplorickettsia* and some (but not all) strains of *Nasuia* in *N. virescens* . Furthermore, the scarcity of *Nasuia* in our other Cicadellidae samples could suggest that it might have been replaced by mycetocyte‐associated yeast‐like symbionts, as previously reported in some Deltocephalinae species (Kobiałka et al. 2016; Wu et al. 2023), although this would need experimental validation using fungal 18S rRNA gene sequencing.

Secondary co‐occurring endosymbionts, which have been described before *Diplorickettsia*, and *Sodalis* (Ishii et al. 2013; Kobiałka et al. 2016; Noda et al. 2012), were present in most samples (Figure 4; Table S4). Among those, ***Diplorickettsia*** and ***Sodalis*** exhibited similar complementary patterns as what was described for *Nasuia*, potentially assisting *Sulcia* in amino acid provisioning in specific host contexts. *Diplorickettsia* generally co‐occurred with *Sulcia* and *Nasuia* in *N. virescens* . Similarly, *Sodalis* and *Rickettsia* showed a comparable pattern in *Cofana spectra*, their presence associating with *Sulcia* strain ASV_7, suggesting this *Sulcia* strain might not fully meet the nutritional requirements alone. However, in sample ‘CP07’ where ASV_1 of *Sulcia* is present instead, the absence of those two endosymbionts might indicate strain‐related variability in the ability of *Sulcia* alone to supply host diet. Likewise, the previously mentioned absence of *Diplorickettsia* from sample ‘CP03’ highlights variability, suggesting these secondary symbionts are not universally obligate, but are rather context‐dependent partners whose complementarity might rely on the specific *Sulcia* strain present. While this is speculation, it is an interesting hypothesis to explore.

We also observed potential complementary interactions involving other secondary symbionts, notably ***Wolbachia*** and the facultative symbiont Can. Lariskella. *Wolbachia* was consistently abundant in host species such as *Exitianus* sp., potentially acting as a co‐primary symbiont with *Sulcia*. However, due to the limited sample size and lack of functional data, we refrain from interpreting this as evidence of a co‐obligate role. While *Wolbachia* is known to provide nutritional benefits in the common bedbug *Cimex lectularius* (Hickin et al. 2022), it is more commonly characterised as a reproductive manipulator in arthropods. Further investigation is necessary to determine its role in leafhopper hosts. In some non‐parasitised hosts dominated by *Sulcia*, secondary symbionts appeared largely unnecessary, except for cases like sample CN27, where Can. Endoecteiascidia appeared established. Additionally, the reduced relative abundance of *Sulcia* alongside increased abundances of *Wolbachia* or Can. Lariskella in specific *Stirellus* samples (CN23, CP21) might reflect recent symbiotic acquisitions (Matsuura et al. 2012), although this remains speculative.

Parasitoid bacterial communities differed markedly from their Cicadellidae hosts. **Rhodobacteraceae** and **Comamonadaceae** were significantly enriched in Dryinidae parasitoids relative to hosts and Halictophagidae parasitoids (Table 1). Both bacterial families have previously been detected in diverse insects, including other parasitoid wasps and bees (Badrulisham et al. 2022; Cerqueira et al. 2024; Gómez‐Govea et al. 2024). Conversely, ***Wolbachia*** dominated bacterial communities in Halictophagidae parasitoids, aligning with observations of reproductive symbiont dominance in Strepsiptera and their fruit fly hosts (Towett‐Kirui et al. 2023).

Overall, our findings align well with existing literature, highlighting the conserved association of *Sulcia* with Cicadellidae, and underscoring the complex but potentially context‐dependent roles of co‐occurring symbionts like *Nasuia*, *Diplorickettsia*, *Rickettsia*, and *Sodalis*.

### 4.2 The Effect of Parasitisation on the Microbiota

Parasitoids rely heavily on hosts as their primary resource for nutrition, growth, and survival (Zhou et al. 2022). Parasitoid wasps, in particular, can induce host immune responses or introduce microbial communities that alter host microbiota (Gwokyalya et al. 2024; Oliver et al. 2003). For example, Gwokyalya et al. (2024) reported that parasitism by the wasp *Diachasmimorpha longicaudata* significantly perturbed the gut microbiota of fruit flies, favouring colonisation by harmful bacteria. In contrast, our study found no significant differences in overall bacterial community composition between parasitised and non‐parasitised Cicadellidae hosts (Table 1). Nevertheless, we explored the potential co‐occurrence of intracellular symbionts between hosts and parasitoids, based on shared ASV identity (Figure 5). Although the limited number of specimens prevents firm conclusions regarding stable horizontal transfer, persistent ASV co‐occurrence suggests potential horizontal transmission events or recent infections, warranting further genomic and experimental investigation.

Two symbionts, ***Arsenophonus*** and ***Wolbachia***, showed notable co‐occurrences between Cicadellidae hosts and Halictophagidae parasitoids. *Arsenophonus*, previously reported from various insects including parasitoid wasps, aphids, and leafhoppers (Bressan 2014; Bressan et al. 2012; Kobiałka et al. 2016; Nováková et al. 2009), was detected here in both parasitised and non‐parasitised *Exitianus* sp., with shared ASVs identified between host‐parasitoid pairs (CP08‐PA08, CP10‐PA10; Figure 5). While experimental evidence has shown *Arsenophonus* transferring between hosts and parasitoids in aphid‐wasp systems (Heidari Latibari et al. 2023), confirmation of transfer in our system requires further study. Similarly, *Wolbachia* ASVs were shared between Cicadellidae hosts (*Exitianus* sp. and *Goniagnathus punctifer*) and Halictophagidae parasitoids (pairs CP08‐PA08, CP10‐PA10, CP14‐PA14, CP15‐PA15, CP30‐PA30; Figure 5). For a subset of these samples (pairs CP14‐PA14, CP15‐PA15 and CP30‐PA30), we were also able to retrieve gene sequences of *Wolbachia* surface protein (WSP). In all instances the gene sequences were identical within the pairs of hosts and parasitoids (data not shown). Interestingly, *Wolbachia* showed higher relative abundance in parasitised individuals in four out of six parasitised hosts of *G. punctifer* (CP26, CP28, CP29, CP30; Figure 4), suggesting a possible link between parasitisation and increased *Wolbachia* load. This observation is purely the result of a correlation, however limited by sample size. Although *Wolbachia* DNA detection could potentially result from transient contamination, residual parasitoid tissue, or incomplete parasitoid egg clearance, previous studies have provided robust evidence of such horizontal transmission (Gloder et al. 2021; Vavre et al. 1999; Zhao et al. 2021). Given that our COI sequencing results do not indicate significant DNA contamination (no double peaks in the chromatograms), bacterial contamination between host and parasitoid appears unlikely, further supporting the possibility of recent infections.

***Diplorickettsia* and *Rickettsia*** also occurred in both leafhopper hosts and parasitoids, indicating potential shared infection events. Specifically, *Rickettsia* ASV_35 was identified in *Cofana spectra* and its associated Halictophagidae parasitoid, while two *Diplorickettsia* ASVs were shared between *Nephotettix virescens* and Dryinidae parasitoids (Figure 5). This represents the first detection of *Diplorickettsia* in Dryinidae, although alternative explanations such as transient presence due to undigested parasitoid gut contents must be acknowledged. *Diplorickettsia* and *Rickettsia* have been previously reported from Cicadellidae hosts (e.g., *Diplorickettsia* in *Macrosteles sexnotatus*, Ishii et al. 2013) and parasitoids (e.g., Rickettsia in *Neochrysocharis formosa*, Paulson et al. 2014), with horizontal transmission hypothesised in planthoppers and strepsipteran parasitoids (Hughes et al. 2004; Liu et al. 2024; Noda et al. 2012). These combined observations emphasise *Diplorickettsia* and *Rickettsia* as important targets for future horizontal symbiont transmission studies. Lastly, ***Sodalis*** was consistently detected in both parasitised and non‐parasitised *Cofana spectra* hosts as well as a Halictophagidae parasitoid larva (Figures 4 and 5). Its comparable abundance across parasitised and non‐parasitised hosts indicates a stable association, likely independent of parasitisation status. *Sodalis* is known for both horizontal and vertical transmission across diverse insect groups, including Auchenorrhyncha leafhoppers (Kobiałka, Michalik, Walczak, and Szklarzewicz 2018; Nishino et al. 2016; Renoz et al. 2024; Rubin et al. 2018; Salazar et al. 2021), and serves important nutritional and developmental roles in other insects such as weevils and solitary bees (Kucuk 2020; Rubin et al. 2018). While our results do not clarify the functional role of *Sodalis* in *C. spectra*, its consistent presence suggests a persistent rather than transient symbiotic relationship.

Overall, our study provides new insights into microbial interactions between Cicadellidae and their parasitoids, highlighting notable secondary symbiont associations. Observations of shared ASVs of *Arsenophonus*, *Wolbachia*, *Diplorickettsia*, *Rickettsia*, and *Sodalis* highlight potential horizontal transmission and emphasise the stable association between *Sulcia* and Cicadellidae hosts, alongside more dynamic secondary symbiont interactions. These findings set a foundation for targeted experimental validation and further exploration of symbiont dynamics in host‐parasitoid systems.

## Author Contributions

**Sophany Phauk:** investigation, writing – original draft, methodology, validation, visualization, formal analysis, data curation, resources, writing – review and editing, project administration. **Lorenzo Assentato:** conceptualization, methodology, validation, visualization, writing – review and editing, supervision. **Seanghun Meas:** validation, writing – review and editing, supervision. **Olle Terenius:** investigation, validation, methodology, writing – review and editing, supervision, funding acquisition.

## Conflicts of Interest

The authors declare no conflicts of interest.

## Supporting information


**Figure S1:** Phylogenetic tree of parasitic Dryinidae and Halictophagidae classified into 5 DCG groups and 4 HCG groups interacting between parasites and hosts. A maximum‐likelihood tree with 1000 bootstraps of 23 parasitic samples used in the analysis, based on a 658 bp portion of the mitochondrial cytochrome oxidase subunit I (COI). The analysis was made in Molecular Evolutionary Genetics Analysis (MEGA 11).
**Figure S2:** Positive Control: two samples from the ZymoBIOMICS Microbial Community Standard (Zymo Research) were used as positive controls to evaluate our protocol. When processed along the same pipeline together with the samples from this study, 6 out of 8 bacterial strains were identified correctly at the genus level. *Salmonella enterica* was classified only down to the Family level as Enterobacteriaceae, while *Bacillus subtilis* was undetected. Most of the bacteria had proportions close to the expected ones (14%), with *Listeria* being the main under‐represented, and *Enterococcus* being slightly over‐represented. This might indicate a bias in the community composition skewed towards Enterococcacea.
**Figure S3:** Bacterial community composition of host Cicadellidae and their parasitoid (Dryinidae and Halictophagidae) at the Phylum **(A)** and Family **(B)** levels. ‘NA’ indicates unclassified bacterial taxa.

**Table S1:** Metadata and information of the dataset.
**Table S2:** Read count tracking sequence reads of Parasites of the Illumina Miseq (V3‐V4) amplicons.
**Table S3:** Relative abundance (sequence reads) of bacterial communities in association with Hosts (Hemiptera) and Parasites (Hymenoptera and Strepsiptera).
**Table S4:** Symbiont bacteria associated with Cicadellidae species.

**Table S5:** Illumina MiSeq Primers (341F/805R) of 50 different barcoding primer pairs.

## Data Availability

The raw bacterial 16S rRNA (V3‐V4) gene sequence reads were deposited in the European Nucleotide Archive (ENA accession *PRJEB70784*).
