# Supplementary material for: Primary and Secondary Symbionts of Cambodian Cicadellidae and the Role of Parasitisation
Source: Environ Microbiol Rep. 2025 Sep 16;17(5):e70196. doi: 10.1111/1758-2229.70196 (PMC12440678; doi:10.1111/1758-2229.70196)
Supplement: Supplementary file 1 — Figure S1: Phylogenetic tree of parasitic Dryinidae and Halictophagidae classified into 5 DCG groups and 4 HCG groups interacting between parasites and hosts. A maximum‐likelihood tree with 1000 bootstraps of 23 parasitic samples used in the analysis, based on a 658 bp portion of the mitochondrial cytochrome oxidase subunit I (COI). The analysis was made in Molecular Evolutionary Genetics Analysis (MEGA 11). Figure S2: Positive Control: two samples from the ZymoBIOMICS Microbial Community Standard (Zymo Research) were used as positive controls to evaluate our protocol. When processed along the same pipeline together with the samples from this study, 6 out of 8 bacterial strains were identified correctly at the genus level. Salmonella enterica was classified only down to the Family level as Enterobacteriaceae, while Bacillus subtilis was undetected. Most of the bacteria had proportions close to the expected ones (14%), with Listeria being the main under‐represented, and Enterococcus being slightly over‐represented. This might indicate a bias in the community composition skewed towards Enterococcacea. Figure S3: Bacterial community composition of host Cicadellidae and their parasitoid (Dryinidae and Halictophagidae) at the Phylum (A) and Family (B) levels. ‘NA’ indicates unclassified bacterial taxa. [file EMI4-17-e70196-s003.docx]

**Primary and secondary symbionts of Cambodian Cicadellidae and the role of parasitization**

**Sophany Phauk^1,2^, Lorenzo Assentato^2^, Seanghun Meas^1^ and Olle Terenius^2,^***

^1^ Department of Biology, Faculty of Science, Royal University of Phnom Penh, Cambodia

^2^ Department of Cell and Molecular Biology, Microbiology, Uppsala University, Uppsala, Sweden

* Correspondence. Email address: [olle.terenius@icm.uu.se](mailto:olle.terenius@icm.uu.se). ORCID: <https://orcid.org/0000-0002-9909-1859>

**
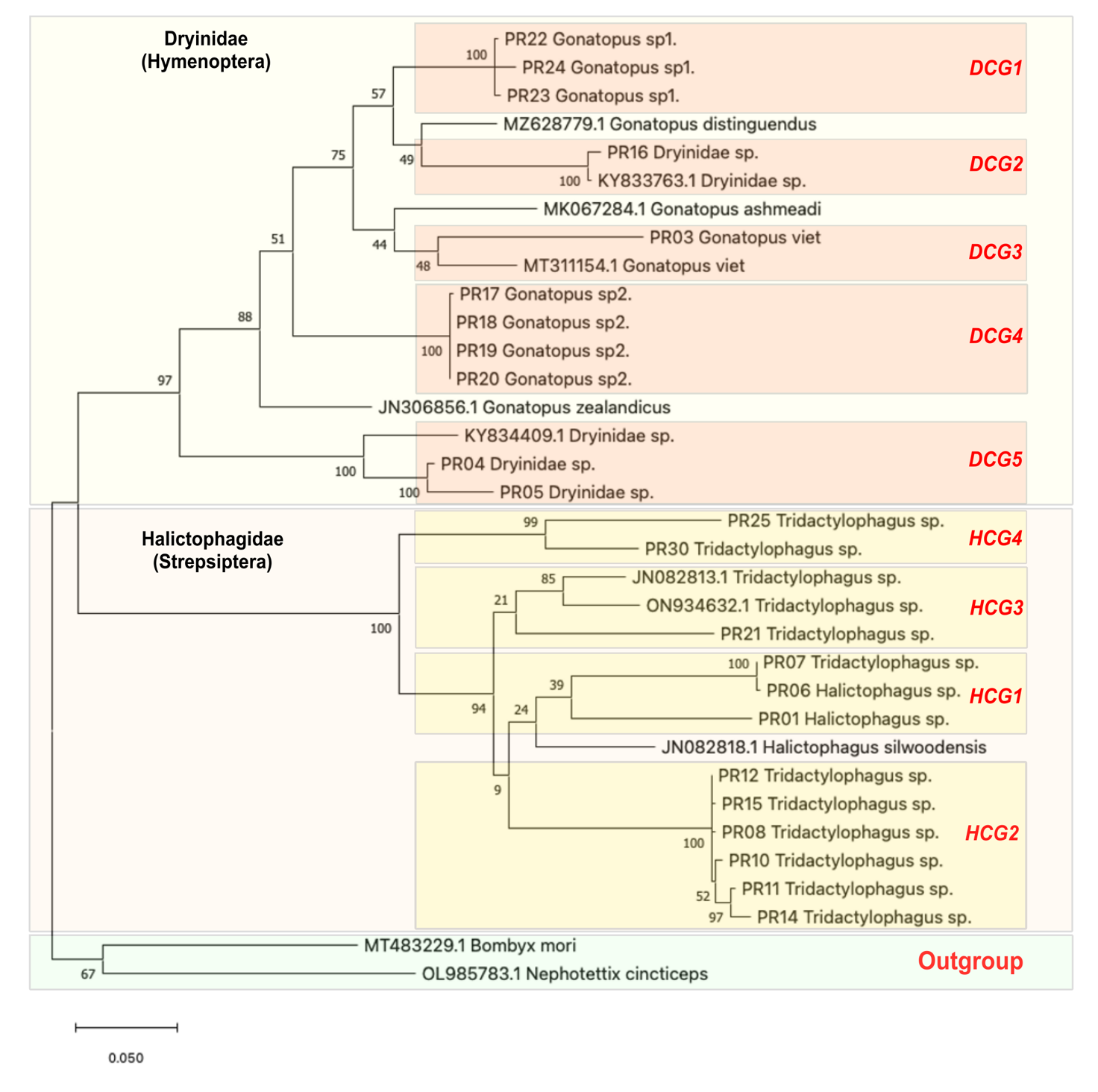
**

**Figure S1:** Phylogenetic tree of parasitic Dryinidae and Halictophagidae were classified into 5 DCG groups and 4 HCG groups interaction between parasites and hosts Cicadellidae. A maximum-likelihood tree with 1,000 bootstraps of 23 parasitic samples used in the analysis, based on a 658 bp portion of the mitochondrial cytochrome oxidase subunit I (COI). The analysis was made in Molecular Evolutionary Genetics Analysis (MEGA 11 vrs).


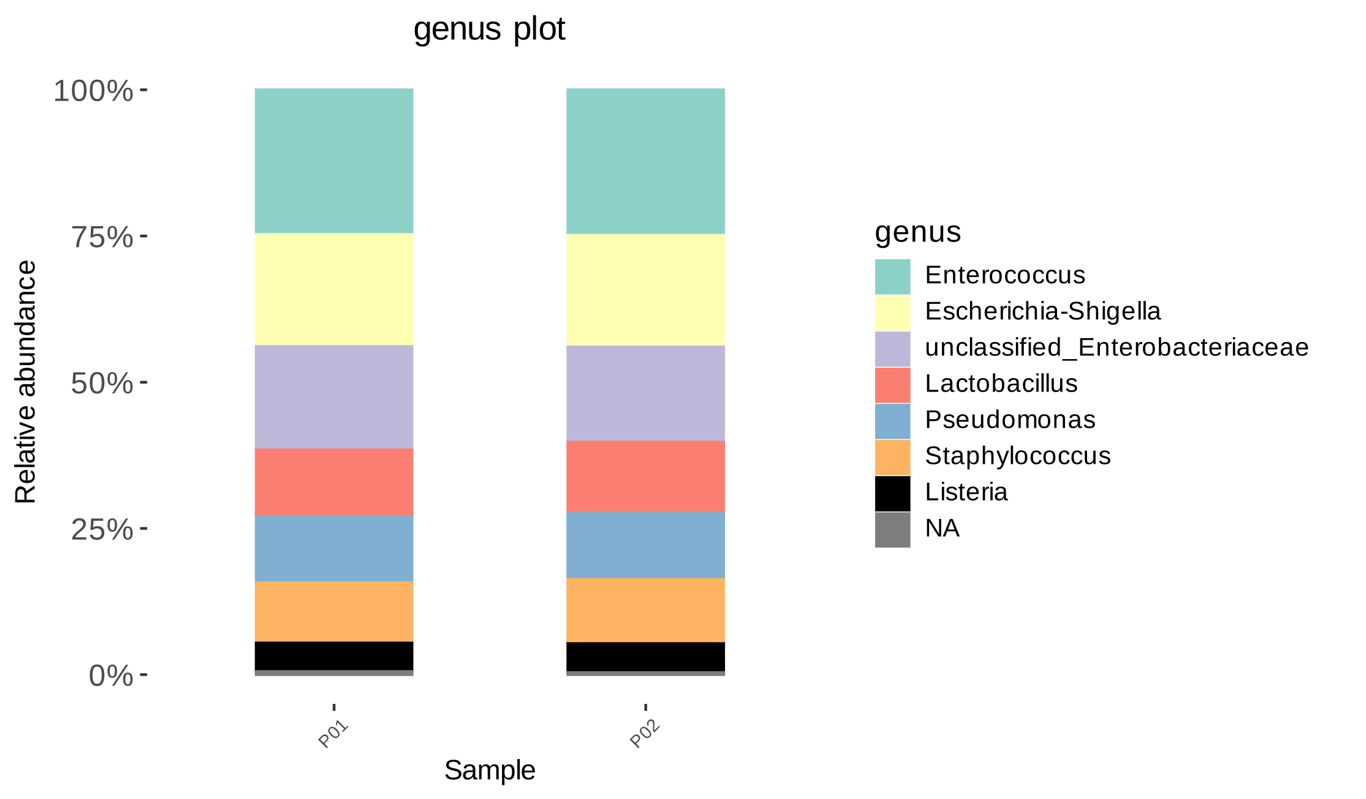


**Figure S2.** Positive Control: two samples from the ZymoBIOMICS Microbial Community Standard (Zymo Research) were used as positive controls to evaluate our protocol. When processed along the same pipeline together with the samples from this study, 6 out of 8 bacterial strains were identified correctly at the genus level. *Salmonella enterica* was classified only down to the Family level as *Enterobacteriaceae*, while *Bacillus subtilis* was undetected. Most of the bacteria had proportions close to the expected ones (14%), with *Listeria* being the main under-represented, and *Enterococcus* being slightly over-represented. This might indicate a bias in the community composition skewed towards *Enterococcacea*

**
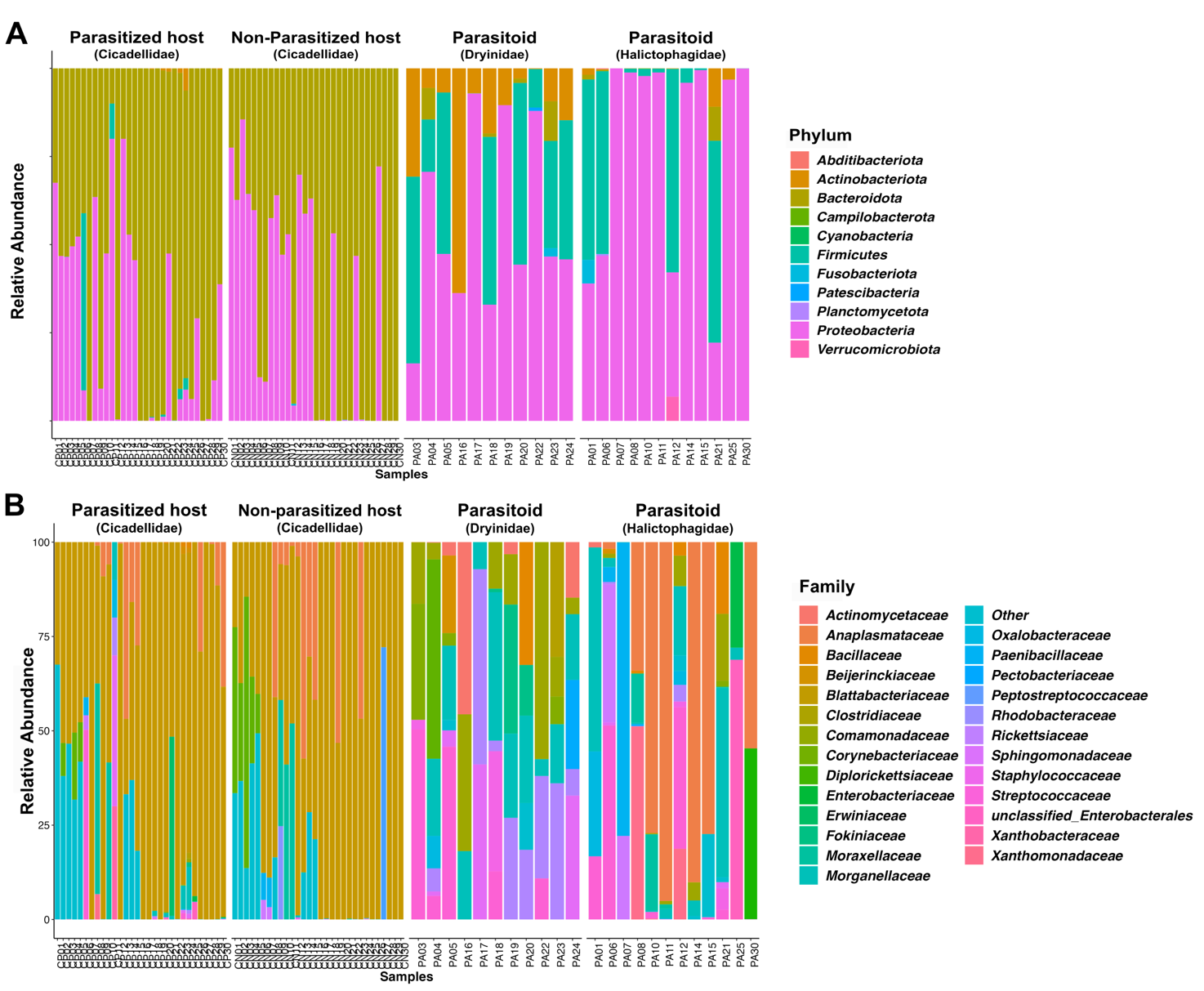
**

**Figure S3:** Bacterial community composition of host Cicadellidae and their parasitoid (Dryinidae and Halictophagidae) at the Phylum **(A)** and Family **(B)** levels. “NA” indicates unclassified bacterial taxa at the genus level.
