# Supplementary material for: Primary and Secondary Symbionts of Cambodian Cicadellidae and the Role of Parasitisation
Source: Environ Microbiol Rep. 2025 Sep 16;17(5):e70196. doi: 10.1111/1758-2229.70196 (PMC12440678; doi:10.1111/1758-2229.70196)
Supplement: Supplementary file 2 — Table S1: Metadata and information of the dataset. Table S2: Read count tracking sequence reads of Parasites of the Illumina Miseq (V3‐V4) amplicons. Table S3: Relative abundance (sequence reads) of bacterial communities in association with Hosts (Hemiptera) and Parasites (Hymenoptera and Strepsiptera). Table S4: Symbiont bacteria associated with Cicadellidae species. [file EMI4-17-e70196-s002.docx]

**Primary and secondary symbionts of Cambodian Cicadellidae and the role of parasitization**

**Sophany Phauk^1,2^, Lorenzo Assentato^2^, Seanghun Meas^1^ and Olle Terenius^2,^***

^1^ Department of Biology, Faculty of Science, Royal University of Phnom Penh, Cambodia

^2^ Department of Cell and Molecular Biology, Microbiology, Uppsala University, Uppsala, Sweden

* Correspondence. Email address: [olle.terenius@icm.uu.se](mailto:olle.terenius@icm.uu.se). ORCID: <https://orcid.org/0000-0002-9909-1859>

**Table S1:** Metadata and information of the dataset

| **SampleID** | **InsectSP** | **Order** | **Sample Type** | **Host Sex** | **Sampling Site** | **Province** | **Location** | **Season** | **Coordination (GPS)** | **Collection date** |
| --- | --- | --- | --- | --- | --- | --- | --- | --- | --- | --- |
| CN01 | *Nephotettix virescens* | Hemiptera | Normal host | M | NSP01 | Kampong Chhnang | Tonle Sap | Dry | 12°11'11.50"N; 104°39'47.30"E | 20-10-2019 |
| CN02 | *Nephotettix virescens* | Hemiptera | Normal host | F | NSP01 | Kampong Chhnang | Tonle Sap | Dry | 12°11'11.50"N; 104°39'47.30"E | 20-10-2019 |
| CN03 | *Nephotettix virescens* | Hemiptera | Normal host | M | NSP02 | Kampong Chhnang | Tonle Sap | Wet | 12°17'3.10"N; 104°35'14.90"E | 20-09-2020 |
| CN04 | *Nephotettix virescens* | Hemiptera | Normal host | F | NSP05 | Battambang | Tonle Sap | Dry | 13°13'9.87"N; 103° 6'5.43"E | 20-01-2020 |
| CN05 | *Nephotettix virescens* | Hemiptera | Normal host | F | NSP05 | Battambang | Tonle Sap | Dry | 13°13'9.87"N; 103° 6'5.43"E | 20-01-2020 |
| CN06 | *Cofana spectra* | Hemiptera | Normal host | M | NSP05 | Battambang | Tonle Sap | Dry | 13°13'9.87"N; 103° 6'5.43"E | 20-01-2020 |
| CN07 | *Cofana spectra* | Hemiptera | Normal host | F | NSP09 | Kampong Thom | Tonle Sap | Wet | 12°53'15.20"N; 104°36'18.80"E | 20-11-2019 |
| CN08 | *Exitianus* sp*.* | Hemiptera | Normal host | M | CSP06 | Kampong Speur | Chambok | Wet | 11°24'9.00"N; 104° 6'38.60"E | 20-10-2019 |
| CN09 | *Exitianus* sp*.* | Hemiptera | Normal host | F | CSP06 | Kampong Speur | Chambok | Wet | 11°24'9.00"N; 104° 6'38.60"E | 20-10-2019 |
| CN10 | *Exitianus* sp*.* | Hemiptera | Normal host | F | CSP07 | Kampong Speur | Chambok | Wet | 11°23'49.80"N; 104° 5'54.60"E | 20-10-2019 |
| CN11 | *Exitianus* sp*.* | Hemiptera | Normal host | F | CSP11 | Kampong Speur | Chambok | Dry | 11°23'29.00"N; 104° 6'25.90"E | 20-01-2020 |
| CN12 | *Exitianus* sp*.* | Hemiptera | Normal host | M | CSP11 | Kampong Speur | Chambok | Dry | 11°23'29.00"N; 104° 6'25.90"E | 20-01-2020 |
| CN13 | *Exitianus* sp*.* | Hemiptera | Normal host | F | NSP02 | Kampong Chhnang | Tonle Sap | Wet | 12°17'3.10"N; 104°35'14.90"E | 20-09-2020 |
| CN14 | *Exitianus* sp*.* | Hemiptera | Normal host | F | NSP04 | Pursat | Tonle Sap | Wet | 12°31'36.30"N; 104° 4'44.85"E | 20-09-2020 |
| CN15 | *Exitianus* sp*.* | Hemiptera | Normal host | F | NSP04 | Pursat | Tonle Sap | Wet | 12°31'36.30"N; 104° 4'44.85"E | 20-09-2020 |
| CN16 | *Maiestas dorsalis* | Hemiptera | Normal host | F | NSP04 | Pursat | Tonle Sap | Wet | 12°31'36.30"N; 104° 4'44.85"E | 20-09-2020 |
| CN17 | *Maiestas dorsalis* | Hemiptera | Normal host | F | NSP06 | Battambang | Tonle Sap | Dry | 13°19'27.50"N; 103° 3'50.00"E | 20-01-2020 |
| CN18 | *Maiestas dorsalis* | Hemiptera | Normal host | M | NSP06 | Battambang | Tonle Sap | Wet | 13°19'27.50"N; 103° 3'50.00"E | 20-09-2020 |
| CN19 | *Maiestas dorsalis* | Hemiptera | Normal host | F | NSP10 | Kampong Thom | Tonle Sap | Wet | 12°47'20.40"N; 104°50'15.20"E | 20-09-2020 |
| CN20 | *Maiestas dorsalis* | Hemiptera | Normal host | M | NSP10 | Kampong Thom | Tonle Sap | Wet | 12°47'20.40"N; 104°50'15.20"E | 20-09-2020 |
| CN21 | *Stirellus* sp. | Hemiptera | Normal host | M | NSP01 | Kampong Chhnang | Tonle Sap | Wet | 12°11'11.50"N; 104°39'47.30"E | 20-07-2020 |
| CN22 | *Stirellus* sp. | Hemiptera | Normal host | F | NSP01 | Kampong Chhnang | Tonle Sap | Wet | 12°11'11.50"N; 104°39'47.30"E | 20-09-2020 |
| CN23 | *Stirellus* sp. | Hemiptera | Normal host | F | NSP07 | Siem Reap | Tonle Sap | Dry | 13°25'35.60"N; 103°45'6.00"E | 20-01-2020 |
| CN24 | *Stirellus* sp. | Hemiptera | Normal host | M | NSP09 | Kampong Thom | Tonle Sap | Dry | 12°53'15.20"N; 104°36'18.80"E | 20-01-2020 |
| CN25 | *Goniagnathus punctifer* | Hemiptera | Normal host | F | NSP04 | Pursat | Tonle Sap | Wet | 12°31'36.30"N; 104° 4'44.85"E | 20-07-2020 |
| CN26 | *Goniagnathus punctifer* | Hemiptera | Normal host | F | NSP04 | Pursat | Tonle Sap | Wet | 12°31'36.30"N; 104° 4'44.85"E | 20-07-2020 |
| CN27 | *Goniagnathus punctifer* | Hemiptera | Normal host | F | NSP04 | Pursat | Tonle Sap | Wet | 12°31'36.30"N; 104° 4'44.85"E | 20-09-2020 |
| CN28 | *Goniagnathus punctifer* | Hemiptera | Normal host | F | NSP09 | Kampong Thom | Tonle Sap | Wet | 12°53'15.20"N; 104°36'18.80"E | 20-07-2020 |
| CN29 | *Goniagnathus punctifer* | Hemiptera | Normal host | F | NSP10 | Kampong Thom | Tonle Sap | Wet | 12°47'20.40"N; 104°50'15.20"E | 20-07-2020 |
| CN30 | *Goniagnathus punctifer* | Hemiptera | Normal host | M | NSP10 | Kampong Thom | Tonle Sap | Wet | 12°47'20.40"N; 104°50'15.20"E | 20-07-2020 |
| CP01 | *Nephotettix virescens* | Hemiptera | Infected host | M | NSP01 | Kampong Chhnang | Tonle Sap | Dry | 12°11'11.50"N; 104°39'47.30"E | 20-01-2020 |
| CP02 | *Nephotettix virescens* | Hemiptera | Infected host | F | NSP01 | Kampong Chhnang | Tonle Sap | Dry | 12°11'11.50"N; 104°39'47.30"E | 20-01-2020 |
| CP03 | *Nephotettix virescens* | Hemiptera | Infected host | M | NSP02 | Kampong Chhnang | Tonle Sap | Wet | 12°17'3.10"N; 104°35'14.90"E | 20-09-2020 |
| CP04 | *Nephotettix virescens* | Hemiptera | Infected host | F | NSP05 | Battambang | Tonle Sap | Dry | 13°13'9.87"N; 103° 6'5.43"E | 20-01-2020 |
| CP05 | *Nephotettix virescens* | Hemiptera | Infected host | F | NSP05 | Battambang | Tonle Sap | Dry | 13°13'9.87"N; 103° 6'5.43"E | 20-01-2020 |
| CP06 | *Cofana spectra* | Hemiptera | Infected host | M | NSP05 | Battambang | Tonle Sap | Dry | 13°13'9.87"N; 103° 6'5.43"E | 20-01-2020 |
| CP07 | *Cofana spectra* | Hemiptera | Infected host | F | NSP09 | Kampong Thom | Tonle Sap | Wet | 12°53'15.20"N; 104°36'18.80"E | 20-11-2019 |
| CP08 | *Exitianus* sp*.* | Hemiptera | Infected host | M | CSP06 | Kampong Speur | Chambok | Wet | 11°24'9.00"N; 104° 6'38.60"E | 20-10-2019 |
| CP09 | *Exitianus* sp*.* | Hemiptera | Infected host | F | CSP06 | Kampong Speur | Chambok | Wet | 11°24'9.00"N; 104° 6'38.60"E | 20-10-2019 |
| CP10 | *Exitianus* sp*.* | Hemiptera | Infected host | F | CSP07 | Kampong Speur | Chambok | Wet | 11°23'49.80"N; 104° 5'54.60"E | 20-10-2019 |
| CP11 | *Exitianus* sp*.* | Hemiptera | Infected host | F | CSP11 | Kampong Speur | Chambok | Dry | 11°23'29.00"N; 104° 6'25.90"E | 20-01-2020 |
| CP12 | *Exitianus* sp*.* | Hemiptera | Infected host | M | CSP11 | Kampong Speur | Chambok | Dry | 11°23'29.00"N; 104° 6'25.90"E | 20-01-2020 |
| CP13 | *Exitianus* sp*.* | Hemiptera | Infected host | F | NSP02 | Kampong Chhnang | Tonle Sap | Wet | 12°17'3.10"N; 104°35'14.90"E | 20-09-2020 |
| CP14 | *Exitianus* sp*.* | Hemiptera | Infected host | F | NSP04 | Pursat | Tonle Sap | Wet | 12°31'36.30"N; 104° 4'44.85"E | 20-09-2020 |
| CP15 | *Exitianus* sp*.* | Hemiptera | Infected host | F | NSP04 | Pursat | Tonle Sap | Wet | 12°31'36.30"N; 104° 4'44.85"E | 20-09-2020 |
| CP16 | *Maiestas dorsalis* | Hemiptera | Infected host | F | NSP04 | Pursat | Tonle Sap | Wet | 12°31'36.30"N; 104° 4'44.85"E | 20-09-2020 |
| CP17 | *Maiestas dorsalis* | Hemiptera | Infected host | F | NSP06 | Battambang | Tonle Sap | Dry | 13°19'27.50"N; 103° 3'50.00"E | 20-01-2020 |
| CP18 | *Maiestas dorsalis* | Hemiptera | Infected host | M | NSP06 | Battambang | Tonle Sap | Wet | 13°19'27.50"N; 103° 3'50.00"E | 20-09-2020 |
| CP19 | *Maiestas dorsalis* | Hemiptera | Infected host | F | NSP10 | Kampong Thom | Tonle Sap | Wet | 12°47'20.40"N; 104°50'15.20"E | 20-09-2020 |
| CP20 | *Maiestas dorsalis* | Hemiptera | Infected host | M | NSP10 | Kampong Thom | Tonle Sap | Wet | 12°47'20.40"N; 104°50'15.20"E | 20-09-2020 |
| CP21 | *Stirellus* sp. | Hemiptera | Infected host | M | NSP01 | Kampong Chhnang | Tonle Sap | Wet | 12°11'11.50"N; 104°39'47.30"E | 20-07-2020 |
| CP22 | *Stirellus* sp. | Hemiptera | Infected host | F | NSP01 | Kampong Chhnang | Tonle Sap | Wet | 12°11'11.50"N; 104°39'47.30"E | 20-09-2020 |
| CP23 | *Stirellus* sp. | Hemiptera | Infected host | F | NSP07 | Siem Reap | Tonle Sap | Dry | 13°25'35.60"N; 103°45'6.00"E | 20-01-2020 |
| CP24 | *Stirellus* sp. | Hemiptera | Infected host | M | NSP09 | Kampong Thom | Tonle Sap | Dry | 12°53'15.20"N; 104°36'18.80"E | 20-01-2020 |
| CP25 | *Goniagnathus punctifer* | Hemiptera | Infected host | M | NSP04 | Pursat | Tonle Sap | Wet | 12°31'36.30"N; 104° 4'44.85"E | 20-07-2020 |
| CP26 | *Goniagnathus punctifer* | Hemiptera | Infected host | M | NSP04 | Pursat | Tonle Sap | Wet | 12°31'36.30"N; 104° 4'44.85"E | 20-07-2020 |
| CP27 | *Goniagnathus punctifer* | Hemiptera | Infected host | F | NSP04 | Pursat | Tonle Sap | Wet | 12°31'36.30"N; 104° 4'44.85"E | 20-09-2020 |
| CP28 | *Goniagnathus punctifer* | Hemiptera | Infected host | F | NSP09 | Kampong Thom | Tonle Sap | Wet | 12°53'15.20"N; 104°36'18.80"E | 20-07-2020 |
| CP29 | *Goniagnathus punctifer* | Hemiptera | Infected host | F | NSP10 | Kampong Thom | Tonle Sap | Wet | 12°47'20.40"N; 104°50'15.20"E | 20-07-2020 |
| CP30 | *Goniagnathus punctifer* | Hemiptera | Infected host | M | NSP10 | Kampong Thom | Tonle Sap | Wet | 12°47'20.40"N; 104°50'15.20"E | 20-07-2020 |
| PA01 | *Halictophagidae G3* | Strepsiptera | Parasitized | - | NSP01 | Kampong Chhnang | Tonle Sap | Dry | 12°11'11.50"N; 104°39'47.30"E | 20-01-2020 |
| PA03 | *Gonatopus viet G3* | Hymenoptera | Parasitized | - | NSP02 | Kampong Chhnang | Tonle Sap | Dry | 12°17'3.10"N; 104°35'14.90"E | 20-01-2020 |
| PA04 | *Dryinidae* sp*. G5* | Hymenoptera | Parasitized | - | NSP05 | Battambang | Tonle Sap | Dry | 13°13'9.87"N; 103° 6'5.43"E | 20-01-2020 |
| PA05 | *Dryinidae* sp*. G5* | Hymenoptera | Parasitized | - | NSP05 | Battambang | Tonle Sap | Dry | 13°13'9.87"N; 103° 6'5.43"E | 20-01-2020 |
| PA06 | *Halictophagidae G3* | Strepsiptera | Parasitized | - | NSP05 | Battambang | Tonle Sap | Dry | 13°13'9.87"N; 103° 6'5.43"E | 20-01-2020 |
| PA07 | *Halictophagidae G3* | Strepsiptera | Parasitized | - | NSP09 | Kampong Thom | Tonle Sap | Wet | 12°53'15.20"N; 104°36'18.80"E | 20-11-2019 |
| PA08 | *Halictophagidae G4* | Strepsiptera | Parasitized | - | CSP06 | Kampong Speur | Chambok | Wet | 11°24'9.00"N; 104° 6'38.60"E | 20-10-2019 |
| PA10 | *Halictophagidae G4* | Strepsiptera | Parasitized | - | CSP07 | Kampong Speur | Chambok | Wet | 11°23'49.80"N; 104° 5'54.60"E | 20-10-2019 |
| PA11 | *Halictophagidae G4* | Strepsiptera | Parasitized | - | CSP11 | Kampong Speur | Chambok | Dry | 11°23'29.00"N; 104° 6'25.90"E | 20-01-2020 |
| PA12 | *Halictophagidae G4* | Strepsiptera | Parasitized | - | CSP11 | Kampong Speur | Chambok | Dry | 11°23'29.00"N; 104° 6'25.90"E | 20-01-2020 |
| PA14 | *Halictophagidae G4* | Strepsiptera | Parasitized | - | NSP04 | Pursat | Tonle Sap | Wet | 12°31'36.30"N; 104° 4'44.85"E | 20-09-2020 |
| PA15 | *Halictophagidae G4* | Strepsiptera | Parasitized | - | NSP04 | Pursat | Tonle Sap | Wet | 12°31'36.30"N; 104° 4'44.85"E | 20-09-2020 |
| PA16 | *Dryinidae* sp*. G2* | Hymenoptera | Parasitized | - | NSP04 | Pursat | Tonle Sap | Wet | 12°31'36.30"N; 104° 4'44.85"E | 20-09-2020 |
| PA17 | *Gonatopus* sp2*. G3* | Hymenoptera | Parasitized | - | NSP06 | Battambang | Tonle Sap | Dry | 13°19'27.50"N; 103° 3'50.00"E | 20-01-2020 |
| PA18 | *Gonatopus* sp2*. G3* | Hymenoptera | Parasitized | - | NSP06 | Battambang | Tonle Sap | Wet | 13°19'27.50"N; 103° 3'50.00"E | 20-09-2020 |
| PA19 | *Gonatopus* sp2*. G3* | Hymenoptera | Parasitized | - | NSP10 | Kampong Thom | Tonle Sap | Wet | 12°47'20.40"N; 104°50'15.20"E | 20-09-2020 |
| PA20 | *Gonatopus* sp2*. G3* | Hymenoptera | Parasitized | - | NSP10 | Kampong Thom | Tonle Sap | Wet | 12°47'20.40"N; 104°50'15.20"E | 20-09-2020 |
| PA21 | *Halictophagidae G2* | Strepsiptera | Parasitized | - | NSP01 | Kampong Chhnang | Tonle Sap | Wet | 12°11'11.50"N; 104°39'47.30"E | 20-07-2020 |
| PA22 | *Gonatopus* sp1*. G1* | Hymenoptera | Parasitized | - | NSP01 | Kampong Chhnang | Tonle Sap | Wet | 12°11'11.50"N; 104°39'47.30"E | 20-09-2020 |
| PA23 | *Gonatopus* sp1*. G1* | Hymenoptera | Parasitized | - | NSP07 | Siem Reap | Tonle Sap | Dry | 13°25'35.60"N; 103°45'6.00"E | 20-01-2020 |
| PA24 | *Gonatopus* sp1*. G1* | Hymenoptera | Parasitized | - | NSP09 | Kampong Thom | Tonle Sap | Dry | 12°53'15.20"N; 104°37'56.75"E | 20-01-2020 |
| PA25 | *Halictophagidae G1* | Strepsiptera | Parasitized | - | NSP04 | Pursat | Tonle Sap | Wet | 12°31'36.30"N; 104° 4'44.85"E | 20-07-2020 |
| PA30 | *Halictophagidae G1* | Strepsiptera | Parasitized | - | NSP10 | Kampong Thom | Tonle Sap | Wet | 12°47'20.40"N; 104°50'15.20"E | 20-07-2020 |

**Table S2**: Read count tracking sequence reads by Parasites of the Illumina Miseq (V3-V4) amplicons

| **No.** | **SampleID** | **InsectSP** | **dada2_input** | **filtered** | **dada_f** | **dada_r** | **merged** | **nonchim** | **percentage**  **reads_retained** |
| --- | --- | --- | --- | --- | --- | --- | --- | --- | --- |
| 1 | CN01 | *Nephotettix virescens* | 10104 | 10091 | 10061 | 10025 | 9881 | 8523 | 84.4 |
| 2 | CN02 | *Nephotettix virescens* | 6346 | 6335 | 6295 | 6279 | 6204 | 5773 | 91.0 |
| 3 | CN03 | *Nephotettix virescens* | 15100 | 15081 | 15040 | 14982 | 14713 | 13373 | 88.6 |
| 4 | CN04 | *Nephotettix virescens* | 20190 | 20166 | 20095 | 20064 | 19836 | 17486 | 86.6 |
| 5 | CN05 | *Nephotettix virescens* | 24930 | 24889 | 24831 | 24790 | 24542 | 21480 | 86.2 |
| 6 | CN06 | *Cofana spectra* | 31453 | 31411 | 31365 | 31352 | 31030 | 27120 | 86.2 |
| 7 | CN07 | *Cofana spectra* | 39764 | 39712 | 39667 | 39619 | 39182 | 37115 | 93.3 |
| 8 | CN08 | *Exitianus* sp*.* | 13818 | 13798 | 13735 | 13706 | 13646 | 12690 | 91.8 |
| 9 | CN09 | *Exitianus* sp*.* | 44040 | 43986 | 43820 | 43716 | 43042 | 41567 | 94.4 |
| 10 | CN10 | *Exitianus* sp*.* | 3216 | 3214 | 3210 | 3205 | 3179 | 3072 | 95.5 |
| 11 | CN11 | *Exitianus* sp*.* | 14538 | 14521 | 14501 | 14491 | 14372 | 13852 | 95.3 |
| 12 | CN12 | *Exitianus* sp*.* | 522 | 521 | 511 | 509 | 504 | 485 | 92.9 |
| 13 | CN13 | *Exitianus* sp*.* | 3411 | 3408 | 3397 | 3391 | 3380 | 3227 | 94.6 |
| 14 | CN14 | *Exitianus* sp*.* | 24759 | 24717 | 24675 | 24644 | 24562 | 23324 | 94.2 |
| 15 | CN15 | *Exitianus* sp*.* | 17325 | 17303 | 17262 | 17225 | 17172 | 15880 | 91.7 |
| 16 | CN16 | *Maiestas dorsalis* | 7547 | 7535 | 7500 | 7499 | 7082 | 6739 | 89.3 |
| 17 | CN17 | *Maiestas dorsalis* | 1034 | 1033 | 1028 | 1027 | 966 | 890 | 86.1 |
| 18 | CN18 | *Maiestas dorsalis* | 81 | 81 | 78 | 78 | 72 | 66 | 81.5 |
| 19 | CN19 | *Maiestas dorsalis* | 62 | 62 | 62 | 62 | 61 | 59 | 95.2 |
| 20 | CN20 | *Maiestas dorsalis* | 14 | 14 | 14 | 14 | 13 | 13 | 92.9 |
| 21 | CN21 | *Stirellus* sp. | 958 | 957 | 944 | 943 | 938 | 899 | 93.8 |
| 22 | CN22 | *Stirellus* sp. | 12417 | 12387 | 12360 | 12359 | 12309 | 11705 | 94.3 |
| 23 | CN23 | *Stirellus* sp. | 15460 | 15437 | 15407 | 15404 | 15312 | 14616 | 94.5 |
| 24 | CN24 | *Stirellus* sp. | 9159 | 9149 | 9107 | 9098 | 9009 | 8433 | 92.1 |
| 25 | CN25 | *Goniagnathus punctifer* | 16506 | 16486 | 16400 | 16437 | 16309 | 15209 | 92.1 |
| 26 | CN26 | *Goniagnathus punctifer* | 4758 | 4745 | 4715 | 4726 | 4685 | 4348 | 91.4 |
| 27 | CN27 | *Goniagnathus punctifer* | 12412 | 12401 | 12361 | 12332 | 12275 | 11904 | 95.9 |
| 28 | CN28 | *Goniagnathus punctifer* | 6687 | 6677 | 6643 | 6640 | 6566 | 6417 | 96.0 |
| 29 | CN29 | *Goniagnathus punctifer* | 18112 | 18088 | 18075 | 18056 | 17981 | 17156 | 94.7 |
| 30 | CN30 | *Goniagnathus punctifer* | 12422 | 12404 | 12382 | 12375 | 12324 | 11694 | 94.1 |
| 31 | CP01 | *Nephotettix virescens* | 3931 | 3923 | 3905 | 3888 | 3862 | 3282 | 83.5 |
| 32 | CP02 | *Nephotettix virescens* | 15418 | 15400 | 15362 | 15325 | 15141 | 12723 | 82.5 |
| 33 | CP03 | *Nephotettix virescens* | 1488 | 1486 | 1478 | 1472 | 1373 | 1146 | 77.0 |
| 34 | CP04 | *Nephotettix virescens* | 12313 | 12288 | 12258 | 12228 | 12074 | 10839 | 88.0 |
| 35 | CP05 | *Nephotettix virescens* | 2853 | 2847 | 2843 | 2833 | 2804 | 2445 | 85.7 |
| 36 | CP06 | *Cofana spectra* | 26270 | 26230 | 26052 | 26112 | 25849 | 22895 | 87.2 |
| 37 | CP07 | *Cofana spectra* | 24878 | 24843 | 24773 | 24792 | 23620 | 21335 | 85.8 |
| 38 | CP08 | *Exitianus* sp*.* | 27811 | 27761 | 27643 | 27585 | 27422 | 25380 | 91.3 |
| 39 | CP09 | *Exitianus* sp*.* | 23841 | 23806 | 23745 | 23701 | 23314 | 21723 | 91.1 |
| 40 | CP10 | *Exitianus* sp*.* | 8247 | 8233 | 8215 | 8205 | 8133 | 7638 | 92.6 |
| 41 | CP11 | *Exitianus* sp*.* | 27 | 26 | 21 | 17 | 11 | 10 | 37.0 |
| 42 | CP12 | *Exitianus* sp*.* | 11254 | 11235 | 11200 | 11175 | 11069 | 9955 | 88.5 |
| 43 | CP13 | *Exitianus* sp*.* | 5266 | 5257 | 5248 | 5159 | 5143 | 4576 | 86.9 |
| 44 | CP14 | *Exitianus* sp*.* | 11387 | 11372 | 11354 | 11339 | 11308 | 10507 | 92.3 |
| 45 | CP15 | *Exitianus* sp*.* | 22993 | 22960 | 22901 | 22878 | 22786 | 21412 | 93.1 |
| 46 | CP16 | *Maiestas dorsalis* | 11771 | 11752 | 11688 | 11693 | 11035 | 10005 | 85.0 |
| 47 | CP17 | *Maiestas dorsalis* | 15942 | 15918 | 15872 | 15839 | 14976 | 13099 | 82.2 |
| 48 | CP18 | *Maiestas dorsalis* | 2793 | 2786 | 2751 | 2743 | 2708 | 2215 | 79.3 |
| 49 | CP19 | *Maiestas dorsalis* | 21737 | 21707 | 21610 | 21600 | 20602 | 18720 | 86.1 |
| 50 | CP20 | *Maiestas dorsalis* | 630 | 629 | 604 | 605 | 579 | 528 | 83.8 |
| 51 | CP21 | *Stirellus* sp. | 29063 | 29017 | 28948 | 28962 | 28870 | 25736 | 88.6 |
| 52 | CP22 | *Stirellus* sp. | 4856 | 4849 | 4829 | 4822 | 4802 | 4280 | 88.1 |
| 53 | CP23 | *Stirellus* sp. | 6902 | 6893 | 5900 | 4583 | 4164 | 1755 | 25.4 |
| 54 | CP24 | *Stirellus* sp. | 1087 | 1084 | 831 | 752 | 613 | 422 | 38.8 |
| 55 | CP25 | *Goniagnathus punctifer* | 14373 | 14347 | 14274 | 14274 | 13812 | 12120 | 84.3 |
| 56 | CP26 | *Goniagnathus punctifer* | 9747 | 9734 | 9675 | 9696 | 9594 | 8607 | 88.3 |
| 57 | CP27 | *Goniagnathus punctifer* | 4307 | 4300 | 4289 | 4293 | 4126 | 3758 | 87.3 |
| 58 | CP28 | *Goniagnathus punctifer* | 78931 | 78813 | 78740 | 78659 | 78335 | 73786 | 93.5 |
| 59 | CP29 | *Goniagnathus punctifer* | 11367 | 11348 | 11336 | 11316 | 11282 | 10234 | 90.0 |
| 60 | CP30 | *Goniagnathus punctifer* | 10274 | 10262 | 10236 | 10224 | 10157 | 9364 | 91.1 |
| 61 | PA01 | *Halictophagidae G3* | 18019 | 17993 | 17925 | 17881 | 17588 | 17252 | 95.7 |
| 62 | PA03 | *Gonatopus viet G3* | 16754 | 16733 | 16665 | 16653 | 13018 | 12723 | 75.9 |
| 63 | PA04 | *Dryinidae* sp*. G5* | 21251 | 21219 | 21113 | 21073 | 20738 | 20206 | 95.1 |
| 64 | PA05 | *Dryinidae* sp*. G5* | 23519 | 23482 | 23310 | 23253 | 21221 | 20918 | 88.9 |
| 65 | PA06 | *Halictophagidae G3* | 30173 | 30133 | 29778 | 29341 | 28124 | 27541 | 91.3 |
| 66 | PA07 | *Halictophagidae G3* | 21936 | 21903 | 21782 | 21721 | 19727 | 18862 | 86.0 |
| 67 | PA08 | *Halictophagidae G4* | 30285 | 30238 | 30103 | 30075 | 29585 | 28963 | 95.6 |
| 68 | PA10 | *Halictophagidae G4* | 30834 | 30790 | 30654 | 30683 | 30075 | 29258 | 94.9 |
| 69 | PA11 | *Halictophagidae G4* | 11560 | 11542 | 11492 | 11475 | 11216 | 11087 | 95.9 |
| 70 | PA12 | *Halictophagidae G4* | 11563 | 11542 | 11449 | 11436 | 10987 | 10793 | 93.3 |
| 71 | PA14 | *Halictophagidae G4* | 6697 | 6687 | 6665 | 6679 | 6647 | 6560 | 98.0 |
| 72 | PA15 | *Halictophagidae G4* | 14544 | 14518 | 14477 | 14446 | 14239 | 13967 | 96.0 |
| 73 | PA16 | *Dryinidae* sp*. G2* | 3433 | 3431 | 3430 | 3422 | 3053 | 3016 | 87.9 |
| 74 | PA17 | *Gonatopus* sp2*. G3* | 12933 | 12919 | 12867 | 12853 | 12709 | 12212 | 94.4 |
| 75 | PA18 | *Gonatopus* sp2*. G3* | 16205 | 16182 | 15993 | 16036 | 15186 | 14976 | 92.4 |
| 76 | PA19 | *Gonatopus* sp2*. G3* | 29610 | 29566 | 29314 | 29294 | 28673 | 27801 | 93.9 |
| 77 | PA20 | *Gonatopus* sp2*. G3* | 31955 | 31911 | 31805 | 31716 | 31237 | 30254 | 94.7 |
| 78 | PA21 | *Halictophagidae G2* | 70413 | 70320 | 70115 | 69928 | 69272 | 67985 | 96.6 |
| 79 | PA22 | *Gonatopus* sp1*. G1* | 6513 | 6507 | 6488 | 6478 | 6416 | 6070 | 93.2 |
| 80 | PA23 | *Gonatopus* sp1*. G1* | 16698 | 16675 | 16605 | 16575 | 15293 | 14535 | 87.0 |
| 81 | PA24 | *Gonatopus* sp1*. G1* | 23614 | 23585 | 23432 | 23470 | 23007 | 22569 | 95.6 |
| 82 | PA25 | *Halictophagidae G1* | 17146 | 17133 | 17097 | 17053 | 16968 | 16868 | 98.4 |
| 83 | PA30 | *Halictophagidae G1* | 4219 | 4213 | 4167 | 4142 | 4021 | 3778 | 89.5 |

**Table S3**: Relative abundance (sequence reads) of bacterial communities in association with Host (Hemiptera) and Parasites (Hymenoptera and Strepsiptera)

| **ASVs** | **Phylum** | **Class** | **Order** | **Family** | **Genus** | **ASVs Abundance** |
| --- | --- | --- | --- | --- | --- | --- |
| ASV_1 | Bacteroidota | Bacteroidia | Flavobacteriales | Blattabacteriaceae | Candidatus Sulcia | 49251 |
| ASV_2 | Bacteroidota | Bacteroidia | Flavobacteriales | Blattabacteriaceae | Candidatus Sulcia | 47691 |
| ASV_3 | Proteobacteria | Alphaproteobacteria | Rickettsiales | Anaplasmataceae | Wolbachia | 41638 |
| ASV_4 | Bacteroidota | Bacteroidia | Flavobacteriales | Blattabacteriaceae | Candidatus Sulcia | 40087 |
| ASV_5 | Proteobacteria | Alphaproteobacteria | Rickettsiales | Anaplasmataceae | Wolbachia | 39993 |
| ASV_6 | Bacteroidota | Bacteroidia | Flavobacteriales | Blattabacteriaceae | Candidatus Sulcia | 38674 |
| ASV_7 | Bacteroidota | Bacteroidia | Flavobacteriales | Blattabacteriaceae | Candidatus Sulcia | 33112 |
| ASV_8 | Bacteroidota | Bacteroidia | Flavobacteriales | Blattabacteriaceae | Candidatus Sulcia | 32791 |
| ASV_11 | Bacteroidota | Bacteroidia | Flavobacteriales | Blattabacteriaceae | Candidatus Sulcia | 24216 |
| ASV_12 | Bacteroidota | Bacteroidia | Flavobacteriales | Blattabacteriaceae | Candidatus Sulcia | 23783 |
| ASV_13 | Proteobacteria | Alphaproteobacteria | Rickettsiales | Fokiniaceae | Candidatus Lariskella | 6237 |
| ASV_14 | Proteobacteria | Alphaproteobacteria | Rickettsiales | Fokiniaceae | Candidatus Lariskella | 5972 |
| ASV_16 | Proteobacteria | Gammaproteobacteria | Burkholderiales | Oxalobacteraceae | unclassified_Oxalobacteraceae | 19770 |
| ASV_17 | Bacteroidota | Bacteroidia | Flavobacteriales | Blattabacteriaceae | Candidatus Sulcia | 19072 |
| ASV_18 | Bacteroidota | Bacteroidia | Flavobacteriales | Blattabacteriaceae | Candidatus Sulcia | 18440 |
| ASV_19 | Proteobacteria | Gammaproteobacteria | Burkholderiales | Oxalobacteraceae | Candidatus Nasuia | 18076 |
| ASV_20 | Proteobacteria | Gammaproteobacteria | Burkholderiales | Oxalobacteraceae | Candidatus Nasuia | 18058 |
| ASV_21 | Bacteroidota | Bacteroidia | Flavobacteriales | Blattabacteriaceae | Candidatus Sulcia | 17993 |
| ASV_22 | Bacteroidota | Bacteroidia | Flavobacteriales | Blattabacteriaceae | Candidatus Sulcia | 17340 |
| ASV_25 | Proteobacteria | Gammaproteobacteria | Enterobacterales | Morganellaceae | Arsenophonus | 14182 |
| ASV_26 | Firmicutes | Bacilli | Lactobacillales | Streptococcaceae | Lactococcus | 14050 |
| ASV_27 | Proteobacteria | Gammaproteobacteria | Enterobacterales | Morganellaceae | Arsenophonus | 13532 |
| ASV_28 | Firmicutes | Bacilli | Lactobacillales | Streptococcaceae | Lactococcus | 13276 |
| ASV_29 | Proteobacteria | Alphaproteobacteria | Rhodobacterales | Rhodobacteraceae | unclassified_Rhodobacteraceae | 10811 |
| ASV_30 | Proteobacteria | Gammaproteobacteria | Diplorickettsiales | Diplorickettsiaceae | Diplorickettsia | 10430 |
| ASV_31 | Proteobacteria | Alphaproteobacteria | Rhodobacterales | Rhodobacteraceae | unclassified_Rhodobacteraceae | 10106 |
| ASV_32 | Proteobacteria | Gammaproteobacteria | Diplorickettsiales | Diplorickettsiaceae | Diplorickettsia | 10063 |
| ASV_33 | Proteobacteria | Gammaproteobacteria | Xanthomonadales | Xanthomonadaceae | Xanthomonas | 8244 |
| ASV_34 | Proteobacteria | Gammaproteobacteria | Xanthomonadales | Xanthomonadaceae | Xanthomonas | 7998 |
| ASV_35 | Proteobacteria | Alphaproteobacteria | Rickettsiales | Rickettsiaceae | Rickettsia | 7579 |
| ASV_36 | Proteobacteria | Alphaproteobacteria | Rickettsiales | Rickettsiaceae | Rickettsia | 7504 |
| ASV_37 | Proteobacteria | Gammaproteobacteria | Diplorickettsiales | Diplorickettsiaceae | Diplorickettsia | 7201 |
| ASV_38 | Proteobacteria | Gammaproteobacteria | Diplorickettsiales | Diplorickettsiaceae | Diplorickettsia | 7148 |
| ASV_39 | Proteobacteria | Alphaproteobacteria | Rickettsiales | Anaplasmataceae | Wolbachia | 5403 |
| ASV_40 | Actinobacteriota | Actinobacteria | Corynebacteriales | Corynebacteriaceae | Corynebacterium | 5017 |
| ASV_41 | Proteobacteria | Alphaproteobacteria | Rickettsiales | Anaplasmataceae | Wolbachia | 4927 |
| ASV_43 | Proteobacteria | Gammaproteobacteria | Xanthomonadales | Rhodanobacteraceae | Rhodanobacter | 4596 |
| ASV_45 | Proteobacteria | Gammaproteobacteria | Enterobacterales | Morganellaceae | Arsenophonus | 4256 |
| ASV_46 | Proteobacteria | Gammaproteobacteria | Xanthomonadales | Rhodanobacteraceae | Rhodanobacter | 4232 |
| ASV_48 | Proteobacteria | Gammaproteobacteria | Enterobacterales | Morganellaceae | Arsenophonus | 4041 |
| ASV_49 | Proteobacteria | Gammaproteobacteria | Piscirickettsiales | Piscirickettsiaceae | Candidatus Endoecteinascidia | 4028 |
| ASV_51 | Proteobacteria | Gammaproteobacteria | Piscirickettsiales | Piscirickettsiaceae | Candidatus Endoecteinascidia | 3898 |
| ASV_53 | Proteobacteria | Gammaproteobacteria | Enterobacterales | unclassified_Enterobacterales | NA | 3768 |
| ASV_54 | Proteobacteria | Gammaproteobacteria | Burkholderiales | Comamonadaceae | unclassified_Comamonadaceae | 3665 |
| ASV_55 | Proteobacteria | Gammaproteobacteria | Burkholderiales | Comamonadaceae | unclassified_Comamonadaceae | 3613 |
| ASV_56 | Proteobacteria | Gammaproteobacteria | Pseudomonadales | Moraxellaceae | Acinetobacter | 3523 |
| ASV_58 | Proteobacteria | Gammaproteobacteria | Enterobacterales | unclassified_Enterobacterales | NA | 3353 |
| ASV_59 | Proteobacteria | Alphaproteobacteria | Sphingomonadales | Sphingomonadaceae | Sphingobium | 3330 |
| ASV_60 | Proteobacteria | Gammaproteobacteria | Pseudomonadales | Moraxellaceae | Acinetobacter | 3268 |
| ASV_61 | Firmicutes | Bacilli | Bacillales | Bacillaceae | Bacillus | 3141 |
| ASV_62 | Proteobacteria | Alphaproteobacteria | Sphingomonadales | Sphingomonadaceae | Sphingobium | 3121 |
| ASV_63 | Proteobacteria | Gammaproteobacteria | Enterobacterales | Morganellaceae | Arsenophonus | 3111 |
| ASV_64 | Firmicutes | Bacilli | Bacillales | Bacillaceae | Bacillus | 2977 |
| ASV_65 | Proteobacteria | Gammaproteobacteria | Enterobacterales | Morganellaceae | Arsenophonus | 2835 |
| ASV_68 | Proteobacteria | Gammaproteobacteria | Enterobacterales | unclassified_Enterobacterales | NA | 2547 |
| ASV_69 | Proteobacteria | Gammaproteobacteria | Enterobacterales | Erwiniaceae | unclassified_Erwiniaceae | 2526 |
| ASV_70 | Firmicutes | Bacilli | Lactobacillales | Streptococcaceae | Streptococcus | 2508 |
| ASV_71 | Firmicutes | Bacilli | Bacillales | Bacillaceae | Bacillus | 2497 |
| ASV_72 | Proteobacteria | Alphaproteobacteria | Rhodobacterales | Rhodobacteraceae | Paracoccus | 2424 |
| ASV_73 | Proteobacteria | Gammaproteobacteria | Burkholderiales | Comamonadaceae | unclassified_Comamonadaceae | 2397 |
| ASV_74 | Proteobacteria | Alphaproteobacteria | Rhodobacterales | Rhodobacteraceae | Paracoccus | 2382 |
| ASV_75 | Firmicutes | Bacilli | Lactobacillales | Streptococcaceae | Streptococcus | 2378 |
| ASV_76 | Proteobacteria | Gammaproteobacteria | Enterobacterales | unclassified_Enterobacterales | NA | 2335 |
| ASV_77 | Proteobacteria | Gammaproteobacteria | Enterobacterales | Erwiniaceae | unclassified_Erwiniaceae | 2314 |
| ASV_78 | Firmicutes | Bacilli | Bacillales | Bacillaceae | Bacillus | 2284 |
| ASV_79 | Firmicutes | Bacilli | Paenibacillales | Paenibacillaceae | Paenibacillus | 2278 |
| ASV_80 | Firmicutes | Bacilli | Paenibacillales | Paenibacillaceae | Paenibacillus | 2226 |
| ASV_81 | Proteobacteria | Gammaproteobacteria | Burkholderiales | Comamonadaceae | unclassified_Comamonadaceae | 2220 |
| ASV_84 | Proteobacteria | Alphaproteobacteria | Sphingomonadales | Sphingomonadaceae | unclassified_Sphingomonadaceae | 2028 |
| ASV_85 | Proteobacteria | Gammaproteobacteria | Burkholderiales | Oxalobacteraceae | Massilia | 1929 |
| ASV_86 | Proteobacteria | Gammaproteobacteria | Pseudomonadales | Pseudomonadaceae | Pseudomonas | 1914 |
| ASV_87 | Firmicutes | Bacilli | Lactobacillales | Streptococcaceae | Streptococcus | 1908 |
| ASV_88 | Firmicutes | Bacilli | Lactobacillales | Streptococcaceae | Streptococcus | 1906 |
| ASV_90 | Proteobacteria | Gammaproteobacteria | Burkholderiales | Oxalobacteraceae | Massilia | 1849 |
| ASV_91 | Proteobacteria | Alphaproteobacteria | Sphingomonadales | Sphingomonadaceae | unclassified_Sphingomonadaceae | 1842 |
| ASV_92 | Proteobacteria | Gammaproteobacteria | Enterobacterales | Pectobacteriaceae | Sodalis | 1837 |
| ASV_94 | Proteobacteria | Gammaproteobacteria | Pseudomonadales | Pseudomonadaceae | Pseudomonas | 1821 |
| ASV_95 | Firmicutes | Clostridia | Clostridiales | Clostridiaceae | Proteiniclasticum | 1789 |
| ASV_96 | Proteobacteria | Gammaproteobacteria | Pseudomonadales | Moraxellaceae | Acinetobacter | 1783 |
| ASV_98 | Proteobacteria | Gammaproteobacteria | Enterobacterales | Pectobacteriaceae | Sodalis | 1770 |
| ASV_100 | Firmicutes | Clostridia | Clostridiales | Clostridiaceae | Proteiniclasticum | 1746 |
| ASV_101 | Firmicutes | Bacilli | Lactobacillales | Streptococcaceae | Streptococcus | 1726 |
| ASV_103 | Proteobacteria | Gammaproteobacteria | Enterobacterales | Pectobacteriaceae | Sodalis | 1629 |
| ASV_104 | Proteobacteria | Alphaproteobacteria | Rickettsiales | Anaplasmataceae | Wolbachia | 1605 |
| ASV_105 | Proteobacteria | Gammaproteobacteria | Enterobacterales | Pectobacteriaceae | Sodalis | 1577 |
| ASV_106 | Proteobacteria | Gammaproteobacteria | Pasteurellales | Pasteurellaceae | Haemophilus | 1547 |
| ASV_107 | Actinobacteriota | Actinobacteria | Actinomycetales | Actinomycetaceae | Actinomyces | 1546 |
| ASV_108 | Firmicutes | Bacilli | Lactobacillales | Streptococcaceae | Streptococcus | 1540 |
| ASV_109 | Proteobacteria | Gammaproteobacteria | Pasteurellales | Pasteurellaceae | Haemophilus | 1536 |
| ASV_110 | Firmicutes | Bacilli | Lactobacillales | Carnobacteriaceae | unclassified_Carnobacteriaceae | 1514 |
| ASV_112 | Proteobacteria | Gammaproteobacteria | Pseudomonadales | Moraxellaceae | Acinetobacter | 1454 |
| ASV_113 | Firmicutes | Bacilli | Lactobacillales | Carnobacteriaceae | Granulicatella | 1453 |
| ASV_114 | Firmicutes | Clostridia | Peptostreptococcales-Tissierellales | Peptostreptococcaceae | Romboutsia | 1441 |
| ASV_115 | Actinobacteriota | Actinobacteria | Actinomycetales | Actinomycetaceae | Actinomyces | 1438 |
| ASV_116 | Firmicutes | Bacilli | Lactobacillales | Streptococcaceae | Lactococcus | 1398 |
| ASV_117 | Firmicutes | Bacilli | Lactobacillales | unclassified_Lactobacillales | NA | 1353 |
| ASV_118 | Firmicutes | Clostridia | Peptostreptococcales-Tissierellales | Peptostreptococcaceae | Romboutsia | 1350 |
| ASV_119 | Proteobacteria | Gammaproteobacteria | Enterobacterales | Pectobacteriaceae | Sodalis | 1342 |
| ASV_120 | Proteobacteria | Gammaproteobacteria | Enterobacterales | Pectobacteriaceae | Sodalis | 1328 |
| ASV_121 | Firmicutes | Bacilli | Staphylococcales | Staphylococcaceae | Staphylococcus | 1323 |
| ASV_123 | Firmicutes | Bacilli | Lactobacillales | Streptococcaceae | Streptococcus | 1304 |
| ASV_124 | Firmicutes | Clostridia | Peptostreptococcales-Tissierellales | Peptostreptococcaceae | Romboutsia | 1275 |
| ASV_125 | Firmicutes | Bacilli | Lactobacillales | unclassified_Lactobacillales | NA | 1272 |
| ASV_126 | Firmicutes | Clostridia | Peptostreptococcales-Tissierellales | Peptostreptococcaceae | Romboutsia | 1268 |
| ASV_127 | Firmicutes | Bacilli | Lactobacillales | Streptococcaceae | Lactococcus | 1256 |
| ASV_129 | Proteobacteria | Gammaproteobacteria | Enterobacterales | Pectobacteriaceae | Sodalis | 1218 |
| ASV_130 | Proteobacteria | Gammaproteobacteria | Enterobacterales | Pectobacteriaceae | Sodalis | 1216 |
| ASV_131 | Proteobacteria | Alphaproteobacteria | Rickettsiales | Anaplasmataceae | Wolbachia | 1206 |
| ASV_132 | Proteobacteria | Gammaproteobacteria | Enterobacterales | Pectobacteriaceae | Sodalis | 1203 |
| ASV_133 | Proteobacteria | Gammaproteobacteria | Burkholderiales | Rhodocyclaceae | Dechlorobacter | 1190 |
| ASV_134 | Proteobacteria | Gammaproteobacteria | Burkholderiales | Rhodocyclaceae | Dechlorobacter | 1185 |
| ASV_135 | Firmicutes | Bacilli | Staphylococcales | Staphylococcaceae | Staphylococcus | 1174 |
| ASV_136 | Firmicutes | Bacilli | Lactobacillales | Streptococcaceae | Streptococcus | 1161 |
| ASV_137 | Proteobacteria | Alphaproteobacteria | Rhizobiales | Beijerinckiaceae | Methylobacterium-Methylorubrum | 1051 |
| ASV_138 | Actinobacteriota | Actinobacteria | Micrococcales | Micrococcaceae | unclassified_Micrococcaceae | 1026 |
| ASV_139 | Proteobacteria | Alphaproteobacteria | Rhizobiales | Beijerinckiaceae | Methylobacterium-Methylorubrum | 992 |
| ASV_140 | Proteobacteria | Alphaproteobacteria | Rhizobiales | Rhizobiaceae | Aureimonas | 985 |
| ASV_141 | Actinobacteriota | Actinobacteria | Micrococcales | Microbacteriaceae | unclassified_Microbacteriaceae | 963 |
| ASV_142 | Proteobacteria | Gammaproteobacteria | Pseudomonadales | Moraxellaceae | Acinetobacter | 961 |
| ASV_144 | Bacteroidota | Bacteroidia | Sphingobacteriales | NS11-12 marine group | NA | 925 |
| ASV_145 | Proteobacteria | Gammaproteobacteria | Pseudomonadales | Moraxellaceae | Acinetobacter | 923 |
| ASV_147 | Actinobacteriota | Actinobacteria | Micrococcales | Microbacteriaceae | unclassified_Microbacteriaceae | 909 |
| ASV_148 | Proteobacteria | Gammaproteobacteria | Enterobacterales | Pectobacteriaceae | Sodalis | 900 |
| ASV_149 | Firmicutes | Bacilli | Thermoactinomycetales | Thermoactinomycetaceae | Thermoactinomyces | 892 |
| ASV_150 | Bacteroidota | Bacteroidia | Sphingobacteriales | NS11-12 marine group | NA | 884 |
| ASV_151 | Actinobacteriota | Actinobacteria | Micrococcales | Micrococcaceae | Micrococcus | 881 |
| ASV_152 | Firmicutes | Bacilli | Paenibacillales | Paenibacillaceae | Paenibacillus | 875 |
| ASV_153 | Actinobacteriota | Actinobacteria | Micrococcales | Microbacteriaceae | unclassified_Microbacteriaceae | 874 |
| ASV_155 | Firmicutes | Bacilli | Paenibacillales | Paenibacillaceae | Paenibacillus | 861 |
| ASV_156 | Proteobacteria | Gammaproteobacteria | Xanthomonadales | Xanthomonadaceae | Stenotrophomonas | 858 |
| ASV_158 | Bacteroidota | Bacteroidia | Flavobacteriales | Flavobacteriaceae | Flavobacterium | 852 |
| ASV_159 | Firmicutes | Bacilli | Thermoactinomycetales | Thermoactinomycetaceae | Thermoactinomyces | 851 |
| ASV_160 | Bacteroidota | Bacteroidia | Flavobacteriales | Flavobacteriaceae | Flavobacterium | 842 |
| ASV_161 | Proteobacteria | Alphaproteobacteria | Rhizobiales | Beijerinckiaceae | Methylobacterium-Methylorubrum | 842 |
| ASV_162 | Proteobacteria | Gammaproteobacteria | Enterobacterales | Enterobacteriaceae | unclassified_Enterobacteriaceae | 831 |
| ASV_163 | Firmicutes | Bacilli | Lactobacillales | Streptococcaceae | Streptococcus | 820 |
| ASV_164 | Proteobacteria | Alphaproteobacteria | Rhizobiales | Rhizobiaceae | Aureimonas | 819 |
| ASV_165 | Actinobacteriota | Actinobacteria | Micrococcales | Microbacteriaceae | Curtobacterium | 815 |
| ASV_166 | Proteobacteria | Alphaproteobacteria | Rhizobiales | Beijerinckiaceae | Methylobacterium-Methylorubrum | 813 |
| ASV_167 | Proteobacteria | Gammaproteobacteria | Pseudomonadales | Pseudomonadaceae | Pseudomonas | 811 |
| ASV_168 | Actinobacteriota | Actinobacteria | Propionibacteriales | Propionibacteriaceae | Cutibacterium | 806 |
| ASV_169 | Proteobacteria | Gammaproteobacteria | Pseudomonadales | Pseudomonadaceae | Pseudomonas | 798 |
| ASV_170 | Proteobacteria | Alphaproteobacteria | Sphingomonadales | Sphingomonadaceae | Sphingomonas | 782 |
| ASV_171 | Proteobacteria | Gammaproteobacteria | Xanthomonadales | Xanthomonadaceae | Stenotrophomonas | 760 |
| ASV_172 | Proteobacteria | Gammaproteobacteria | Cellvibrionales | Halieaceae | OM60(NOR5) clade | 758 |
| ASV_175 | Firmicutes | Bacilli | Lactobacillales | Streptococcaceae | Streptococcus | 747 |
| ASV_177 | Actinobacteriota | Actinobacteria | Propionibacteriales | Propionibacteriaceae | Cutibacterium | 720 |
| ASV_178 | Firmicutes | Bacilli | Staphylococcales | Staphylococcaceae | Staphylococcus | 711 |
| ASV_179 | Actinobacteriota | Actinobacteria | Actinomycetales | Actinomycetaceae | Flaviflexus | 686 |
| ASV_181 | Firmicutes | Bacilli | Staphylococcales | Staphylococcaceae | Staphylococcus | 677 |
| ASV_182 | Proteobacteria | Gammaproteobacteria | Cellvibrionales | Halieaceae | OM60(NOR5) clade | 666 |
| ASV_183 | Firmicutes | Bacilli | Lactobacillales | Streptococcaceae | Streptococcus | 651 |
| ASV_184 | Proteobacteria | Alphaproteobacteria | Sphingomonadales | Sphingomonadaceae | Sphingomonas | 619 |
| ASV_185 | Firmicutes | Bacilli | Lactobacillales | Streptococcaceae | Streptococcus | 615 |
| ASV_186 | Firmicutes | Bacilli | Lactobacillales | Leuconostocaceae | Leuconostoc | 601 |
| ASV_187 | Proteobacteria | Gammaproteobacteria | Enterobacterales | Enterobacteriaceae | unclassified_Enterobacteriaceae | 572 |
| ASV_188 | Fusobacteriota | Fusobacteriia | Fusobacteriales | Fusobacteriaceae | Fusobacterium | 564 |
| ASV_189 | Proteobacteria | Alphaproteobacteria | Sphingomonadales | Sphingomonadaceae | Sphingobium | 556 |
| ASV_190 | Firmicutes | Bacilli | Thermoactinomycetales | Thermoactinomycetaceae | Thermoactinomyces | 532 |
| ASV_191 | Firmicutes | Bacilli | Lactobacillales | unclassified_Lactobacillales | NA | 529 |
| ASV_192 | Firmicutes | Clostridia | Clostridiales | Clostridiaceae | Proteiniclasticum | 518 |
| ASV_193 | Fusobacteriota | Fusobacteriia | Fusobacteriales | Fusobacteriaceae | Fusobacterium | 516 |
| ASV_194 | Actinobacteriota | Actinobacteria | Actinomycetales | Actinomycetaceae | Flaviflexus | 516 |
| ASV_195 | Firmicutes | Bacilli | Thermoactinomycetales | Thermoactinomycetaceae | Thermoactinomyces | 507 |
| ASV_196 | Proteobacteria | Alphaproteobacteria | Rhizobiales | Beijerinckiaceae | Methylobacterium-Methylorubrum | 502 |
| ASV_197 | Firmicutes | Bacilli | Lactobacillales | Leuconostocaceae | Leuconostoc | 446 |
| ASV_198 | Proteobacteria | Gammaproteobacteria | Enterobacterales | Pectobacteriaceae | Sodalis | 438 |
| ASV_199 | Proteobacteria | Gammaproteobacteria | Pseudomonadales | Moraxellaceae | Acinetobacter | 431 |
| ASV_200 | Proteobacteria | Gammaproteobacteria | Pasteurellales | Pasteurellaceae | Haemophilus | 424 |
| ASV_201 | Firmicutes | Bacilli | Lactobacillales | unclassified_Lactobacillales | NA | 423 |
| ASV_202 | Actinobacteriota | Actinobacteria | Micrococcales | Micrococcaceae | Rothia | 422 |
| ASV_203 | Firmicutes | Clostridia | Clostridiales | Clostridiaceae | Proteiniclasticum | 415 |
| ASV_204 | Proteobacteria | Gammaproteobacteria | Pseudomonadales | Pseudomonadaceae | Pseudomonas | 413 |
| ASV_205 | Actinobacteriota | Actinobacteria | Micrococcales | Micrococcaceae | Rothia | 412 |
| ASV_206 | Proteobacteria | Gammaproteobacteria | Enterobacterales | Pectobacteriaceae | Sodalis | 405 |
| ASV_207 | Proteobacteria | Alphaproteobacteria | Rhodobacterales | Rhodobacteraceae | unclassified_Rhodobacteraceae | 404 |
| ASV_208 | Proteobacteria | Gammaproteobacteria | Pasteurellales | Pasteurellaceae | Haemophilus | 402 |
| ASV_209 | Proteobacteria | Alphaproteobacteria | Sphingomonadales | Sphingomonadaceae | Sphingobium | 402 |
| ASV_210 | Actinobacteriota | Actinobacteria | Actinomycetales | Actinomycetaceae | Actinomyces | 396 |
| ASV_211 | Proteobacteria | Gammaproteobacteria | Pseudomonadales | Pseudomonadaceae | Pseudomonas | 393 |
| ASV_212 | Firmicutes | Bacilli | Lactobacillales | Streptococcaceae | Streptococcus | 392 |
| ASV_213 | Firmicutes | Negativicutes | Veillonellales-Selenomonadales | Veillonellaceae | Veillonella | 392 |
| ASV_214 | Proteobacteria | Gammaproteobacteria | Pseudomonadales | Moraxellaceae | Acinetobacter | 389 |
| ASV_216 | Proteobacteria | Gammaproteobacteria | Pseudomonadales | Pseudomonadaceae | Pseudomonas | 378 |
| ASV_217 | Proteobacteria | Gammaproteobacteria | Pseudomonadales | Pseudomonadaceae | Pseudomonas | 377 |
| ASV_218 | Firmicutes | Negativicutes | Veillonellales-Selenomonadales | Veillonellaceae | Veillonella | 376 |
| ASV_219 | Actinobacteriota | Actinobacteria | Actinomycetales | Actinomycetaceae | Actinomyces | 345 |
| ASV_220 | Firmicutes | Bacilli | Lactobacillales | Streptococcaceae | Streptococcus | 345 |
| ASV_221 | Proteobacteria | Gammaproteobacteria | Burkholderiales | Oxalobacteraceae | Candidatus Nasuia | 343 |
| ASV_224 | Proteobacteria | Gammaproteobacteria | Burkholderiales | Oxalobacteraceae | unclassified_Oxalobacteraceae | 320 |
| ASV_225 | Proteobacteria | Gammaproteobacteria | Legionellales | Legionellaceae | Legionella | 313 |
| ASV_226 | Proteobacteria | Gammaproteobacteria | Legionellales | Legionellaceae | Legionella | 313 |
| ASV_227 | Verrucomicrobiota | Verrucomicrobiae | Verrucomicrobiales | DEV007 | NA | 308 |
| ASV_231 | Actinobacteriota | Actinobacteria | Corynebacteriales | Corynebacteriaceae | Corynebacterium | 291 |
| ASV_232 | Actinobacteriota | Actinobacteria | Actinomycetales | Actinomycetaceae | Actinomyces | 289 |
| ASV_233 | Proteobacteria | Gammaproteobacteria | Burkholderiales | Oxalobacteraceae | unclassified_Oxalobacteraceae | 265 |
| ASV_234 | Firmicutes | Bacilli | Lactobacillales | Streptococcaceae | Streptococcus | 262 |
| ASV_235 | Verrucomicrobiota | Verrucomicrobiae | Verrucomicrobiales | DEV007 | NA | 260 |
| ASV_236 | Proteobacteria | Alphaproteobacteria | Rhizobiales | Beijerinckiaceae | Methylobacterium-Methylorubrum | 234 |
| ASV_237 | Proteobacteria | Alphaproteobacteria | Rhodobacterales | Rhodobacteraceae | unclassified_Rhodobacteraceae | 233 |
| ASV_238 | Proteobacteria | Gammaproteobacteria | Diplorickettsiales | Diplorickettsiaceae | Diplorickettsia | 231 |
| ASV_239 | Firmicutes | Bacilli | Lactobacillales | Streptococcaceae | Streptococcus | 231 |
| ASV_240 | Proteobacteria | Gammaproteobacteria | Burkholderiales | Comamonadaceae | Paucibacter | 231 |
| ASV_241 | Proteobacteria | Alphaproteobacteria | Caulobacterales | Caulobacteraceae | Phenylobacterium | 226 |
| ASV_242 | Proteobacteria | Gammaproteobacteria | Burkholderiales | Burkholderiaceae | Polynucleobacter | 219 |
| ASV_243 | Proteobacteria | Alphaproteobacteria | Caulobacterales | Caulobacteraceae | Phenylobacterium | 215 |
| ASV_244 | Firmicutes | Bacilli | Lactobacillales | Aerococcaceae | Globicatella | 213 |
| ASV_245 | Firmicutes | Bacilli | Paenibacillales | Paenibacillaceae | Paenibacillus | 209 |
| ASV_246 | Firmicutes | Bacilli | Lactobacillales | Aerococcaceae | Globicatella | 206 |
| ASV_247 | Bacteroidota | Bacteroidia | Flavobacteriales | Weeksellaceae | Elizabethkingia | 204 |
| ASV_248 | Firmicutes | Clostridia | Peptostreptococcales-Tissierellales | Anaerovoracaceae | Mogibacterium | 202 |
| ASV_249 | Proteobacteria | Alphaproteobacteria | Rhizobiales | Beijerinckiaceae | Methylobacterium-Methylorubrum | 198 |
| ASV_251 | Proteobacteria | Gammaproteobacteria | Burkholderiales | Comamonadaceae | Paucibacter | 196 |
| ASV_253 | Fusobacteriota | Fusobacteriia | Fusobacteriales | Leptotrichiaceae | Leptotrichia | 194 |
| ASV_254 | Proteobacteria | Gammaproteobacteria | Burkholderiales | Oxalobacteraceae | Candidatus Nasuia | 192 |
| ASV_255 | Proteobacteria | Gammaproteobacteria | Pasteurellales | Pasteurellaceae | Haemophilus | 192 |
| ASV_256 | Firmicutes | Clostridia | Peptostreptococcales-Tissierellales | Anaerovoracaceae | Mogibacterium | 189 |
| ASV_257 | Proteobacteria | Gammaproteobacteria | Burkholderiales | Oxalobacteraceae | Janthinobacterium | 187 |
| ASV_258 | Firmicutes | Bacilli | Lactobacillales | Carnobacteriaceae | unclassified_Carnobacteriaceae | 182 |
| ASV_259 | Proteobacteria | Alphaproteobacteria | Rhodobacterales | Rhodobacteraceae | unclassified_Rhodobacteraceae | 181 |
| ASV_260 | Bacteroidota | Bacteroidia | Flavobacteriales | Weeksellaceae | Elizabethkingia | 179 |
| ASV_261 | Proteobacteria | Gammaproteobacteria | Pseudomonadales | Moraxellaceae | Acinetobacter | 164 |
| ASV_262 | Proteobacteria | Gammaproteobacteria | Pasteurellales | Pasteurellaceae | Haemophilus | 161 |
| ASV_263 | Firmicutes | Bacilli | Staphylococcales | Staphylococcaceae | Staphylococcus | 158 |
| ASV_264 | Actinobacteriota | Actinobacteria | Micrococcales | Microbacteriaceae | unclassified_Microbacteriaceae | 158 |
| ASV_265 | Proteobacteria | Gammaproteobacteria | Burkholderiales | Oxalobacteraceae | Janthinobacterium | 156 |
| ASV_266 | Firmicutes | Bacilli | Lactobacillales | Carnobacteriaceae | unclassified_Carnobacteriaceae | 156 |
| ASV_267 | Actinobacteriota | Actinobacteria | Micrococcales | Microbacteriaceae | unclassified_Microbacteriaceae | 155 |
| ASV_268 | Fusobacteriota | Fusobacteriia | Fusobacteriales | Leptotrichiaceae | Leptotrichia | 155 |
| ASV_270 | Proteobacteria | Gammaproteobacteria | Enterobacterales | Enterobacteriaceae | Cronobacter | 146 |
| ASV_271 | Firmicutes | Bacilli | Lactobacillales | Streptococcaceae | Streptococcus | 145 |
| ASV_272 | Firmicutes | Bacilli | Lactobacillales | Vagococcaceae | Vagococcus | 137 |
| ASV_275 | Firmicutes | Bacilli | Lactobacillales | Vagococcaceae | Vagococcus | 128 |
| ASV_276 | Bacteroidota | Bacteroidia | Flavobacteriales | Crocinitomicaceae | Fluviicola | 124 |
| ASV_278 | Bacteroidota | Bacteroidia | Flavobacteriales | Crocinitomicaceae | Fluviicola | 114 |
| ASV_279 | Bacteroidota | Bacteroidia | Flavobacteriales | Weeksellaceae | Chryseobacterium | 113 |
| ASV_280 | Proteobacteria | Alphaproteobacteria | Rhodobacterales | Rhodobacteraceae | unclassified_Rhodobacteraceae | 109 |
| ASV_281 | Firmicutes | Negativicutes | Veillonellales-Selenomonadales | Veillonellaceae | Veillonella | 107 |
| ASV_282 | Proteobacteria | Gammaproteobacteria | Enterobacterales | Enterobacteriaceae | Cronobacter | 105 |
| ASV_283 | Proteobacteria | Gammaproteobacteria | Cellvibrionales | Halieaceae | OM60(NOR5) clade | 102 |
| ASV_284 | Bacteroidota | Bacteroidia | Flavobacteriales | Weeksellaceae | Chryseobacterium | 96 |
| ASV_285 | Planctomycetota | Planctomycetes | Isosphaerales | Isosphaeraceae | Tundrisphaera | 96 |
| ASV_289 | Firmicutes | Clostridia | Clostridiales | Clostridiaceae | Proteiniclasticum | 88 |
| ASV_290 | Proteobacteria | Gammaproteobacteria | Pseudomonadales | Moraxellaceae | Acinetobacter | 86 |
| ASV_292 | Proteobacteria | Gammaproteobacteria | Pseudomonadales | Moraxellaceae | Acinetobacter | 81 |
| ASV_293 | Firmicutes | Bacilli | Staphylococcales | Staphylococcaceae | Staphylococcus | 77 |
| ASV_297 | Firmicutes | Bacilli | Lactobacillales | Streptococcaceae | Streptococcus | 72 |
| ASV_299 | Firmicutes | Bacilli | Lactobacillales | Streptococcaceae | Lactococcus | 62 |
| ASV_300 | Bacteroidota | Bacteroidia | Chitinophagales | Chitinophagaceae | Flavisolibacter | 61 |
| ASV_301 | Bacteroidota | Bacteroidia | Flavobacteriales | Weeksellaceae | Cloacibacterium | 61 |
| ASV_302 | Firmicutes | Clostridia | Peptostreptococcales-Tissierellales | Peptostreptococcaceae | unclassified_Peptostreptococcaceae | 58 |
| ASV_303 | Firmicutes | Bacilli | Lactobacillales | Carnobacteriaceae | Atopostipes | 57 |
| ASV_305 | Firmicutes | Clostridia | Peptostreptococcales-Tissierellales | Peptostreptococcaceae | unclassified_Peptostreptococcaceae | 54 |
| ASV_306 | Proteobacteria | Alphaproteobacteria | Acetobacterales | Acetobacteraceae | Belnapia | 53 |
| ASV_307 | Proteobacteria | Alphaproteobacteria | Rickettsiales | Anaplasmataceae | Wolbachia | 52 |
| ASV_308 | Bacteroidota | Bacteroidia | Flavobacteriales | Weeksellaceae | Cloacibacterium | 52 |
| ASV_309 | Proteobacteria | Gammaproteobacteria | Aeromonadales | Aeromonadaceae | Aeromonas | 51 |
| ASV_310 | Bacteroidota | Bacteroidia | Chitinophagales | Chitinophagaceae | Flavisolibacter | 50 |
| ASV_312 | Proteobacteria | Alphaproteobacteria | Rhodobacterales | Rhodobacteraceae | Rubellimicrobium | 48 |
| ASV_314 | Firmicutes | Bacilli | Staphylococcales | Staphylococcaceae | Staphylococcus | 45 |
| ASV_315 | Firmicutes | Bacilli | Bacillales | Bacillaceae | Bacillus | 44 |
| ASV_316 | Abditibacteriota | Abditibacteria | Abditibacteriales | Abditibacteriaceae | Abditibacterium | 44 |
| ASV_318 | Firmicutes | Bacilli | Lactobacillales | Carnobacteriaceae | Atopostipes | 42 |
| ASV_319 | Proteobacteria | Alphaproteobacteria | Sphingomonadales | Sphingomonadaceae | unclassified_Sphingomonadaceae | 40 |
| ASV_320 | Proteobacteria | Alphaproteobacteria | Acetobacterales | Acetobacteraceae | Belnapia | 39 |
| ASV_321 | Proteobacteria | Alphaproteobacteria | Sphingomonadales | Sphingomonadaceae | Sphingomonas | 37 |
| ASV_322 | Proteobacteria | Alphaproteobacteria | Rhodobacterales | Rhodobacteraceae | Rubellimicrobium | 36 |
| ASV_323 | Proteobacteria | Gammaproteobacteria | Burkholderiales | Comamonadaceae | unclassified_Comamonadaceae | 36 |
| ASV_324 | Proteobacteria | Gammaproteobacteria | Aeromonadales | Aeromonadaceae | Aeromonas | 35 |
| ASV_326 | Proteobacteria | Gammaproteobacteria | Burkholderiales | Oxalobacteraceae | Massilia | 33 |
| ASV_327 | Proteobacteria | Alphaproteobacteria | Sphingomonadales | Sphingomonadaceae | unclassified_Sphingomonadaceae | 32 |
| ASV_328 | Proteobacteria | Alphaproteobacteria | Sphingomonadales | Sphingomonadaceae | unclassified_Sphingomonadaceae | 31 |
| ASV_329 | Proteobacteria | Alphaproteobacteria | Rickettsiales | Anaplasmataceae | Wolbachia | 29 |
| ASV_330 | Planctomycetota | Planctomycetes | Pirellulales | Pirellulaceae | Pirellula | 29 |
| ASV_331 | Proteobacteria | Gammaproteobacteria | Burkholderiales | Burkholderiaceae | Ralstonia | 28 |
| ASV_332 | Proteobacteria | Gammaproteobacteria | Burkholderiales | Comamonadaceae | unclassified_Comamonadaceae | 28 |
| ASV_333 | Abditibacteriota | Abditibacteria | Abditibacteriales | Abditibacteriaceae | Abditibacterium | 28 |
| ASV_334 | Proteobacteria | Gammaproteobacteria | Burkholderiales | Comamonadaceae | unclassified_Comamonadaceae | 28 |
| ASV_335 | Proteobacteria | Gammaproteobacteria | Burkholderiales | Comamonadaceae | Caenimonas | 26 |
| ASV_336 | Firmicutes | Bacilli | Lactobacillales | Streptococcaceae | Streptococcus | 25 |
| ASV_337 | Campilobacterota | Campylobacteria | Campylobacterales | Campylobacteraceae | Campylobacter | 22 |
| ASV_338 | Proteobacteria | Gammaproteobacteria | Burkholderiales | Oxalobacteraceae | Herbaspirillum | 22 |
| ASV_340 | Proteobacteria | Alphaproteobacteria | Rhizobiales | Devosiaceae | Devosia | 20 |
| ASV_341 | Proteobacteria | Alphaproteobacteria | Rhizobiales | Xanthobacteraceae | unclassified_Xanthobacteraceae | 18 |
| ASV_343 | Proteobacteria | Gammaproteobacteria | Enterobacterales | unclassified_Enterobacterales | NA | 17 |
| ASV_344 | Proteobacteria | Gammaproteobacteria | Pseudomonadales | Moraxellaceae | Acinetobacter | 17 |
| ASV_345 | Campilobacterota | Campylobacteria | Campylobacterales | Campylobacteraceae | Campylobacter | 17 |
| ASV_346 | Proteobacteria | Gammaproteobacteria | Pseudomonadales | Moraxellaceae | Enhydrobacter | 12 |
| ASV_347 | Proteobacteria | Gammaproteobacteria | Burkholderiales | Oxalobacteraceae | Undibacterium | 12 |
| ASV_348 | Patescibacteria | Saccharimonadia | Saccharimonadales | unclassified_Saccharimonadales | NA | 10 |
| ASV_351 | Firmicutes | Bacilli | Lactobacillales | Streptococcaceae | Streptococcus | 7 |
| ASV_352 | Bacteroidota | Bacteroidia | Chitinophagales | Chitinophagaceae | Asinibacterium | 6 |
| ASV_353 | Bacteroidota | Bacteroidia | Chitinophagales | Chitinophagaceae | Asinibacterium | 6 |
| ASV_354 | Proteobacteria | Alphaproteobacteria | Rhizobiales | Rhizobiales Incertae Sedis | Phreatobacter | 5 |
| ASV_356 | Bacteroidota | Bacteroidia | Bacteroidales | Porphyromonadaceae | Porphyromonas | 4 |
| ASV_357 | Cyanobacteria | Vampirivibrionia | Obscuribacterales | Obscuribacteraceae | Candidatus Obscuribacter | 3 |
| ASV_358 | Firmicutes | Bacilli | Lactobacillales | Streptococcaceae | Streptococcus | 3 |
| ASV_359 | Firmicutes | Bacilli | Staphylococcales | Staphylococcaceae | Staphylococcus | 3 |
| ASV_361 | Firmicutes | Bacilli | Lactobacillales | Streptococcaceae | Streptococcus | 2 |
| ASV_363 | Proteobacteria | Gammaproteobacteria | Enterobacterales | Morganellaceae | unclassified_Morganellaceae | 1 |

| Table S4. Symbiont bacteria associated with Cicadellidae species | | | | | |
| --- | --- | --- | --- | --- | --- |
| Cicadellidae host | | | **n** | **Symbionts** | **Parasitoids** |
| Subfamily | **Tribe** | **Species** |  |  |  |
| Cicadellinae | Cicadellini | *Cofana spectra* | 4 | *Sulcia^*^, Sodalis ^(75)^, Rickettsia ^(75)^* | *Halictophagus* sp. (HCG1) |
| Deltocephalinae | Chiasmini | *Exitianus* sp. | 15 | *Sulcia^*^, Arsenophonus ^(33)^, Wolbachia^*^* | *Tridactylophagus* sp3. (HCG2) |
|  |  | *Nephotettix virescens* | 10 | *Sulcia^*^, Nasuia^*^,*  *Diplorickettsia ^(80)^* | *Gonatopus veit* (DCG3), Dryinidae sp2. (DCG5), *Halictophagus* sp. (HCG1) |
|  | Deltocephalini | *Maiestas dorsalis* | 10 | *Sulcia^*^, Wolbachia ^(10)^* | *Dryinidae* sp1. (DCG2), *Gonatopus* sp2. (DCG4) |
|  | Goniagnathini | *Goniagnathus punctifer* | 12 | *Sulcia^*^, Wolbachia ^(50)^,*  *Can.* Endoecteinascidia *^(8)^* | *Tridactylophagus* sp1. (HCG4) |
|  | Stenometopiini | *Stirellus* sp. | 8 | *Sulcia^*^, Wolbachia ^(13)^,*  *Can.* Lariskella *^(13)^* | *Gonatopus* sp1. (DCG1), *Tridactylophagus* sp2. (HCG3) |
| * Symbiont detected in every single sample of the cicadellid host species  ^(%)^ Percentage indicates proportion of samples (out of n) in which the symbiont was detected, based on presence/absence | | | | | |
